# Continuous-flow synthesis of Pd(ii)-anchored amino-functionalised magnetic silica nanoparticles as a robust recyclable catalyst for aqueous Sonogashira cross-coupling

**DOI:** 10.1039/d6ra01007e

**Published:** 2026-03-23

**Authors:** Uthai Sakee, Aekkaphon Mokkarat, Widchaya Radchatawedchakoon, Senee Kruanetr, Ratchaneekorn Pilasombat

**Affiliations:** a Creative Chemistry and Innovation Research Unit, Center of Excellence for Innovation in Chemistry (PERCH-CIC), Department of Chemistry, Faculty of Science, Mahasarakham University Mahasarakham 44150 Thailand uthai.s@msu.ac.th; b Lilly Pharma Co., Ltd No. 1, Soi Ramintra 21, Intersection 20, Ramintra Road, Anusawari, Bang Khen Bangkok 10220 Thailand

## Abstract

A robust and magnetically recoverable palladium nanocatalyst, Fe_3_O_4_@SiO_2_–NH_2_–Pd(ii), was prepared through a scalable continuous-flow coprecipitation/silanisation strategy and evaluated in Sonogashira cross-coupling reactions in water. Although amino-functionalised magnetic silica supports have been reported, their use as heterogeneous catalysts for aqueous Sonogashira coupling has received comparatively limited attention. Under the optimised conditions, using water as the sole reaction medium, CuI/PPh_3_ as the cocatalytic system, and triethylamine as the base, a broad range of iodoanilines and terminal alkynes underwent efficient coupling to afford the desired products in up to 99% isolated yield within short reaction times. The catalyst showed excellent durability and was readily recovered by magnetic separation and reused for at least 15 consecutive cycles with minimal palladium loss, as confirmed by ICP-OES analysis. Hot-filtration and recyclability studies indicate that the reaction proceeds predominantly through a heterogeneous pathway, with at most a minor contribution from soluble palladium species. Hammett analysis revealed clear substituent effects on the reaction rate, providing useful mechanistic insight into the catalytic cycle. The combination of continuous-flow synthesis, aqueous-phase catalysis, and magnetic recyclability highlights the practical potential of this nanocatalyst for more sustainable carbon–carbon bond formation.

## Abstract 2

A robust and magnetically recoverable palladium nanocatalyst, Fe_3_O_4_@SiO_2_–NH_2_–Pd(ii), was prepared through a scalable continuous-flow coprecipitation/silanisation strategy and evaluated in Sonogashira cross-coupling reactions in water.

## Graphical abstract

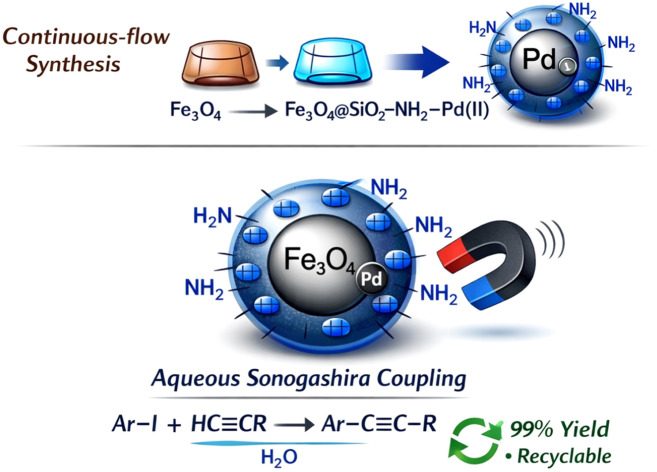

## 1. Introduction

The Sonogashira coupling reaction, a powerful method for constructing carbon–carbon bonds between aryl or vinyl halides and terminal alkynes, has emerged as a cornerstone in modern synthetic organic chemistry.^1–3^ This transformation plays a pivotal role in the synthesis of natural products, pharmaceuticals, agrochemicals, and optoelectronic materials owing to the high reactivity, broad functional group tolerance, and modularity of the coupling partners involved.^4^ Despite these advantages, conventional Sonogashira protocols typically rely on homogeneous palladium catalysts in combination with copper co-catalysts and phosphine ligands, often requiring organic solvents and elevated temperatures. Such conditions raise significant environmental, economic, and operational concerns, particularly with respect to solvent use, catalyst separation and recovery, and the overall sustainability and scalability of Sonogashira processes.^1,5–8^

Previous studies on Pd-based magnetic nanocatalysts for Sonogashira coupling have revealed three recurring limitations: (i) the predominant use of batch synthetic methods, which may compromise reproducibility and limit scalability; (ii) insufficient magnetic separability in some systems, rendering catalyst recovery and reuse less practical; and (iii) continued reliance on organic solvents, thereby undermining the principles of sustainable and green chemistry. Addressing all three challenges within a single catalytic platform remains a key objective in the development of practical heterogeneous Sonogashira catalysts.

Over the past two decades, significant efforts have been devoted to improving the efficiency, selectivity, and sustainability of catalysts used in Sonogashira cross-coupling.^1,6,7,9–11^ Traditional homogeneous systems involving Pd(PPh_3_)_4_ or PdCl_2_(PPh_3_)_2_ combined with copper(i) co-catalysts and amine bases often suffer from poor recyclability, ligand sensitivity, and difficulties in product purification, particularly for pharmaceutical and fine-chemical applications where stringent limits on residual metal contamination are required.^1,5,7,12–14^

To overcome these issues, various heterogeneous catalyst systems have been developed, including palladium nanoparticles immobilised on polymers, carbon materials (*e.g.*, graphene and activated carbon), mesoporous silica (*e.g.*, SBA-15 and MCM-41), metal–organic frameworks (MOFs), covalent organic frameworks (COFs), and magnetic nanoparticles such as Fe_3_O_4_ or γ-Fe_2_O_3_. Among these supports, magnetically separable catalysts are particularly attractive owing to their operational simplicity, efficient recovery using an external magnetic field, and compatibility with the principles of green chemistry.^7,15–19^

Recent advances have highlighted the potential of palladium immobilised on magnetically separable nanostructures for Sonogashira cross-coupling reactions under more sustainable conditions. Various Fe_3_O_4_-based hybrid nanocomposites, including Fe_3_O_4_@SiO_2_, Fe_3_O_4_@graphene oxide, and Fe_3_O_4_@MOFs, have demonstrated promising catalytic activity and reusability. A Pd–Fe_3_O_4_@NH_2_ catalyst was reported to promote Sonogashira and Heck reactions under solvent-free and aqueous conditions with good recyclability.^20^ Likewise, palladium nanoparticles immobilised on monodisperse Fe_3_O_4_@SiO_2_ nanospheres exhibited high catalytic performance and facile magnetic separation.^21^ Pd-loaded Fe_3_O_4_@amine-functionalised graphene oxide nanocomposites prepared *via* sonochemical methods have also shown high turnover frequencies and broad substrate scopes in Sonogashira couplings.^22^ In addition, magnetically recoverable MOF-based nanocatalysts and mesoporous silica-coated Fe_3_O_4_ systems, such as Pd-loaded KIT-6@Fe_3_O_4_, have been successfully applied to Sonogashira, Suzuki, and Ullmann coupling reactions with excellent recyclability and structural stability.^23,24^

Despite considerable progress in the development of magnetically recoverable palladium catalysts for Sonogashira cross-coupling, several limitations remain in currently reported systems. Most Pd-supported magnetic nanocatalysts have been prepared using conventional batch synthetic methods, which often provide limited control over particle size distribution, surface functionalisation, and metal dispersion. These factors can significantly influence catalytic stability and reproducibility, particularly when scaling up catalyst preparation. In addition, many reported systems still rely on organic solvents such as DMSO, DMF, or NMP and operate at relatively high temperatures, which partially offsets the environmental advantages typically associated with heterogeneous catalysis. Furthermore, although numerous Pd-based magnetic catalysts have been reported, detailed mechanistic investigations—particularly studies examining electronic substituent effects and structure–activity relationships—remain relatively scarce, with only limited studies addressing electronic substituent effects and structure–activity relationships in magnetically recoverable Pd systems.^25–29^

Continuous-flow synthesis offers several advantages over traditional batch methods, including improved control of nucleation and growth processes, enhanced reproducibility, and straightforward scalability for nanoparticle preparation. In the context of magnetic nanomaterials, continuous-flow strategies can facilitate uniform surface functionalisation and more controlled metal anchoring, thereby improving catalyst stability during repeated catalytic cycles. However, despite these advantages, continuously synthesised magnetic Pd nanocatalysts have rarely been explored for carbon–carbon cross-coupling reactions conducted under aqueous conditions.

In particular, the integration of continuous-flow nanoparticle synthesis, aqueous-phase Sonogashira catalysis, and systematic mechanistic investigation remains largely unexplored. Addressing these challenges is important for advancing the development of more sustainable heterogeneous catalytic systems capable of combining scalability, environmental compatibility, and mechanistic understanding.

In this work, we report a magnetically recoverable Pd(ii)-anchored nanocatalyst based on amino-functionalised silica-coated magnetite (Fe_3_O_4_@SiO_2_–NH_2_–Pd(ii)) prepared through a scalable continuous-flow strategy. Unlike many previously reported Pd-supported magnetic catalysts that rely on batch synthesis and organic reaction media, the present system integrates continuous-flow preparation of the magnetic support, efficient Sonogashira coupling in water as the sole solvent, and comprehensive mechanistic analysis. The catalytic system enables rapid cross-coupling of iodoaniline derivatives with terminal alkynes under mild aqueous conditions, delivering excellent yields within short reaction times while maintaining high catalytic stability over repeated cycles. In addition to catalytic performance, mechanistic insights were obtained through Hammett analysis, hot-filtration tests, and ICP-OES measurements, providing evidence for predominantly heterogeneous catalysis and revealing clear electronic effects governing reaction kinetics. The integration of scalable catalyst synthesis, green aqueous reaction conditions, and mechanistic evaluation highlights the practical potential of Fe_3_O_4_-based magnetic Pd nanocatalysts for sustainable carbon–carbon bond formation. To the best of our knowledge, this work represents one of the first studies that integrates:

(i) Continuous-flow synthesis of Fe_3_O_4_@SiO_2_–NH_2_ magnetic supports on a gram scale, (ii) Pd(ii) immobilisation through amine coordination without external ligands, and (iii) rapid aqueous Sonogashira coupling accompanied by mechanistic analysis including Hammett correlation, hot-filtration tests, and ICP-OES leaching studies.

The use of 2-iodoaniline as a coupling partner is particularly significant due to its broad applicability in the synthesis of bioactive heterocycles. Products derived from 2-iodoaniline through Sonogashira coupling, such as 2-alkynylaniline intermediates, serve as key building blocks for the construction of indole derivatives *via* subsequent cyclisation reactions. These indole frameworks are associated with diverse pharmacological activities, including anticancer, anti-inflammatory, and antimicrobial properties.^30–32^ For example, Sonogashira coupling products of 2-iodoaniline can undergo ZnBr_2_-mediated or acid-promoted intramolecular cyclisation to afford substituted indoles, quinolines, and other polycyclic heterocycles, highlighting the synthetic versatility of such intermediates.^33–35^

In our previous studies, we reported a robust and scalable continuous-flow strategy for the preparation of Fe_3_O_4_@SiO_2_–NH_2_–Pd(ii) nanoparticles,^36,37^ which provided uniform surface functionalisation and enhanced palladium anchoring, compared with conventional batch methods.^38,39^ Although this material exhibited excellent catalytic performance in reduction reactions, its application in Sonogashira cross-coupling—particularly under aqueous conditions—had not been explored.^40^

## 2. Experimental

### 2.1 Chemicals and materials

Details of all chemicals, reagents, and materials used in this study are provided in the SI (Section S1).

### 2.2 Catalyst preparation

#### 2.2.1 Synthesis of amino-functionalised silica-coated magnetite nanoparticles (Fe_3_O_4_@SiO_2_–NH_2_) – gram-scale preparation (∼10 g)

A modified continuous flow method was applied to synthesize Fe_3_O_4_@SiO_2_–NH_2_ with an expected theoretical yield of approximately 10 g. Aqueous solutions of Fe^2+^ (13.3 mmol, 1.33 g FeSO_4_·7H_2_O) and Fe^3+^ (26.6 mmol, 7.23 g FeCl_3_·6H_2_O) were each dissolved in 50 mL of deionized water and then simultaneously co-injected into a flexible PTFE tubing reactor using two peristaltic pumps (total flow rate = 9 mL min^−1^) at ambient temperature. In parallel, 100 mL of 7.5% aqueous NH_4_OH was injected at the same flow rate to induce co-precipitation, forming magnetite (Fe_3_O_4_) nanoparticles *in situ*.

The freshly formed magnetite was continuously transferred into 500 mL of an ethanol-based coating solution containing tetraethyl orthosilicate (TEOS, 36.0 mmol, 8.1 mL) and 3-aminopropyltriethoxysilane (APTES, 36.0 mmol, 8.5 mL), maintaining a 1 : 1 molar ratio. The suspension was ultrasonicated at 35 kHz and kept at 80 °C for 1 h to promote uniform silica and amine group functionalisation.

After completion, the resulting Fe_3_O_4_@SiO_2_–NH_2_ nanoparticles were magnetically separated using a strong external magnet, thoroughly washed with ethanol (3 × 100 mL), and oven-dried at 80 °C for 24 h. The final dry product was obtained as a black-brown powder with an isolated mass of approximately 9.5 g.

#### 2.2.2 Immobilisation of Pd(ii) onto Fe_3_O_4_@SiO_2_–NH_2_

To synthesize the Pd(ii)-immobilised catalyst on a larger scale, 5.0 g of Fe_3_O_4_@SiO_2_–NH_2_ was suspended in 1250 mL of aqueous PdCl_2_ solution (50 mg L^−1^ Pd) in a 2 L round-bottom flask. The mixture was stirred continuously at ambient temperature for 24 hours to ensure thorough coordination of Pd(ii) ions with the surface amino groups. After completion, the catalyst was magnetically separated, washed thoroughly with absolute ethanol (3 × 100 mL), and dried in an oven at 80 °C overnight. The final material, Fe_3_O_4_@SiO_2_–NH_2_–Pd(ii), was obtained as a dark solid, with the palladium loading determined to be approximately 1.20 wt% by ICP-OES analysis.

### 2.3 General procedure for Sonogashira coupling

#### 2.3.1 General procedure for the optimisation of the Sonogashira coupling reaction

The optimization studies for the Sonogashira coupling of 2-iodoaniline (1a) with phenylacetylene (2a) were carried out in a 10 mL round-bottom flask equipped with a magnetic stir bar under a nitrogen atmosphere. In each experiment, 1a (0.25 mmol), 2a (0.30 mmol, 1.2 equiv.), and Fe_3_O_4_@SiO_2_–NH_2_–Pd(ii) nanocatalyst (variable amount) were suspended in deionized water (2.5 mL). Copper(i) iodide (CuI), triphenylphosphine (PPh_3_), and a selected base were added in the proportions specified for each entry. The mixture was stirred at a given temperature and time, as summarized in Tables 1 and 2.

**Table 1:** Optimisation of catalyst amount and base for the Sonogashira reaction of 2-iodoaniline with phenylacetylene using Fe_3_O_4_@SiO_2_–NH_2_–Pd(ii) as catalyst^a^

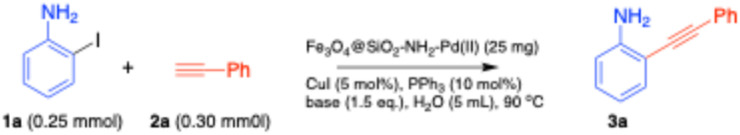				
Entry	Catalyst (mg)	Base	Time (min)	Yield^b^ (%)
1	No Pd catalyst	Et_3_N	60	32
2	5	Et_3_N	20	79
3	10	Et_3_N	15	89
4	15	Et_3_N	10	95
5	20	Et_3_N	7	98
6	25	Et_3_N	5	99
7	30	Et_3_N	5	99
8	25	Pyridine	10	95
9	25	KOH	50	23
10	25	NaOH	50	30
11	25	K_2_CO_3_	20	59
12	25	Na_2_CO_3_	30	34
13	25	NaOAc	90	38
14	25	No base	60	Trace

a Reaction conditions: 2-iodoaniline (0.25 mmol), phenylacetylene (0.30 mmol, 1.2 equiv.), Fe_3_O_4_@SiO_2_–NH_2_–Pd(ii) catalyst (5–30 mg as specified), CuI (5 mol%), PPh_3_ (10 mol%), base (1.5 equiv.), H_2_O (2.5 mL), 90 °C, N_2_ atmosphere. Reaction time as indicated.

b Isolated yield.

**Table 2:** Optimisation of CuI, PPh_3_, and temperature for the Sonogashira reaction of 2-iodoaniline with phenylacetylene using Fe_3_O_4_@SiO_2_–NH_2_–Pd(ii) as catalyst^a^

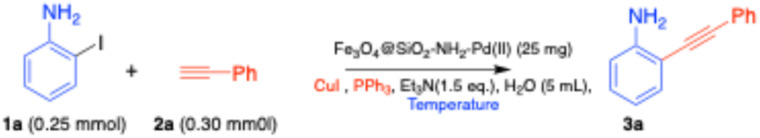					
Entry	CuI (mol%)	PPh_3_ (mol%)	Temp. (°C)	Time (min)	Yield^b^ (%)
1	No CuI	10	90	60	38
2	2.5	10	90	60	59
3	5	10	90	5	99
4	7.5	10	90	5	99
5	5	No PPh_3_	90	60	20
6	5	2.5	90	20	76
7	5	5	90	20	86
8	5	7.5	90	10	92
9	5	10	90	5	99
10	5	12.5	90	5	97
11	5	10	rt	45	30
12	5	10	60	60	60

a Reaction conditions: 2-iodoaniline (0.25 mmol), phenylacetylene (0.30 mmol, 1.2 equiv.), Fe_3_O_4_@SiO_2_–NH_2_–Pd(ii) (25 mg), CuI (0–7.5 mol%), PPh_3_ (0–12.5 mol%), Et_3_N (1.5 equiv.), H_2_O (2.5 mL), N_2_ atmosphere. Reaction time and temperature as indicated.

b Isolated yield.

Upon completion of the reaction (monitored by thin-layer chromatography), the mixture was cooled to room temperature and concentrated under reduced pressure to remove water. The residue was extracted with ethyl acetate (5 × 5 mL), and the catalyst was separated magnetically. The combined organic layers were dried over anhydrous Na_2_SO_4_, filtered, and concentrated under reduced pressure. The crude product was purified by silica gel column chromatography (EtOAc/hexane) to obtain the corresponding coupling product.

All reaction variables, including catalyst loading, base identity, CuI and PPh_3_ loading, and temperature, were systematically varied to determine the optimal conditions (see Tables 1 and 2 for full experimental details and results).

#### 2.3.2 Substrate scope evaluation under optimised conditions

With the optimal reaction conditions established (Section 2.3.1), the generality of the protocol was further explored using various iodoaniline derivatives and terminal alkynes. Each reaction was conducted using 0.25 mmol of the iodoaniline derivative, 1.2 equivalents of the corresponding alkyne, and standard amounts of catalyst, cocatalysts, and base in deionized water under nitrogen.

The reactions were stirred at the previously optimized temperature for the respective durations specified in Table 3. After completion (TLC monitoring), standard workup was performed: aqueous removal under reduced pressure, extraction with ethyl acetate, magnetic separation of the catalyst, drying over Na_2_SO_4_, and chromatographic purification (EtOAc/hexane).

**Table 3:** Sonogashira of iodoaniline derivatives with phenylacetylene derivatives under optimised condition^a^

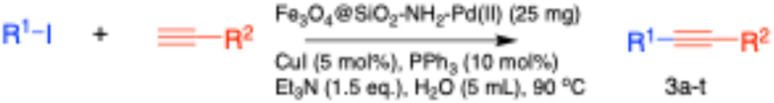						
Entry	R^1^	R^2^	Time (min)	Product, (yield,^b^ %)	TON	TOF (min^−1^)
1	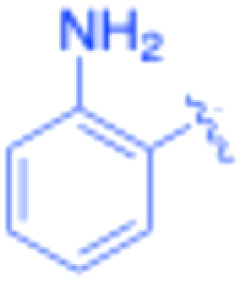	Phenyl	5	3a, 99	85.6	17.12
2	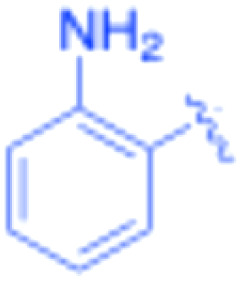	–CH_2_Si(Me)_3_	5	3b, 99	85.6	17.12
3	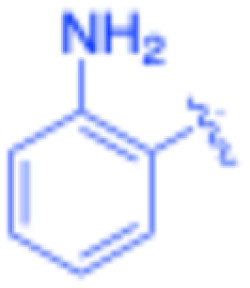	–Si(Me)_3_	5	3c, 99	85.6	17.12
4	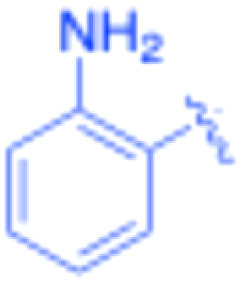	4-Methyl phenyl	10	3d, 97	83.9	8.39
5	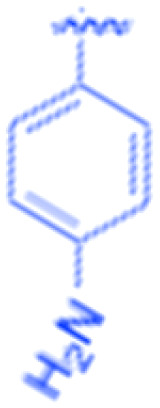	Phenyl	10	3e, 93	80.4	8.04
6	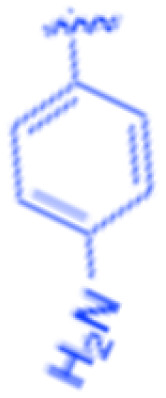	–CH_2_Si(Me)_3_	10	3f, 93	80.4	8.04
7	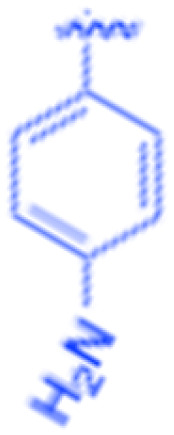	4-Methyl phenyl	10	3g, 94	81.3	8.13
8	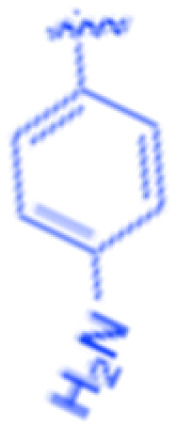	4-Methoxy phenyl	5	3h, 84	72.7	14.54
9	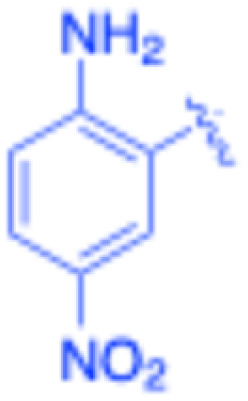	Phenyl	60	3i, 98	84.8	1.41
10	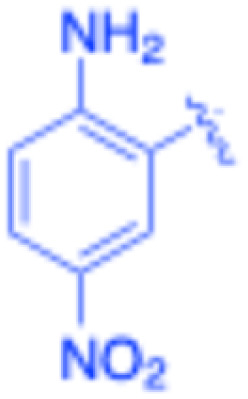	4-Methyl phenyl	45	3j, 94	81.3	1.80
11	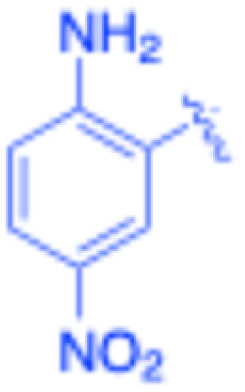	4-Methoxy phenyl	60	3k, 95	82.2	1.37
12	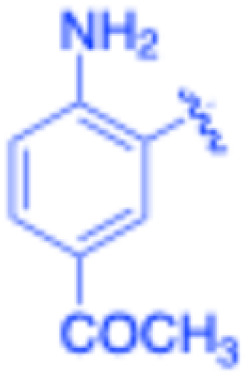	Phenyl	15	3l, 93	80.4	5.36
13	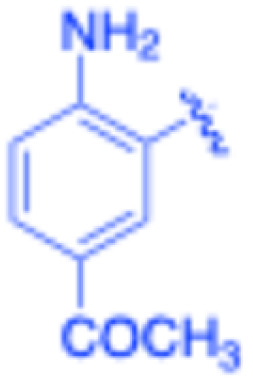	–CH_2_Si(Me)_3_	20	3m, 79	68.3	3.42
14	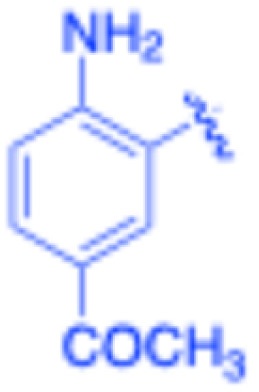	–Si(Me)_3_	20	3n, 75	64.9	3.24
15	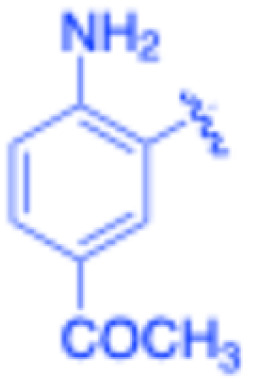	4-Methyl phenyl	5	3o, 98	84.8	16.96
16	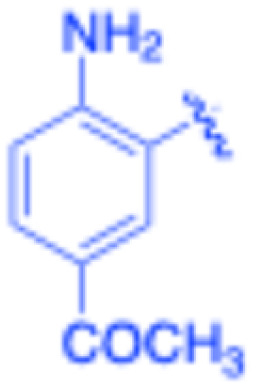	4-Methoxy phenyl	15	3p, 96	83.0	5.53
17	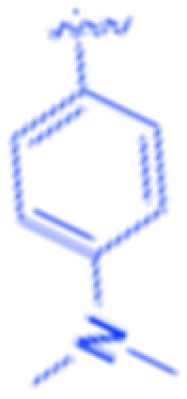	Phenyl	10	3q, 94	81.3	8.13
18	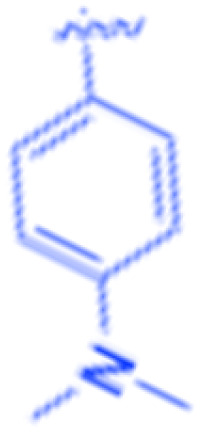	–Si(Me)_3_	5	3r, 89	77.0	15.40
19	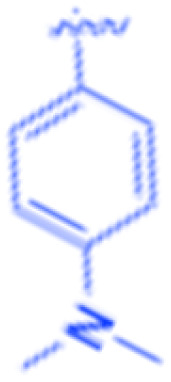	4-Methyl phenyl	5	3s, 83	71.8	14.36
20	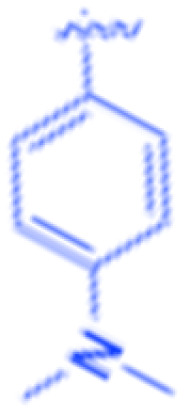	4-Methoxy phenyl	10	3t, 91	78.7	7.87

a Reaction conditions: iodoaniline derivative (0.25 mmol), terminal alkyne (0.30 mmol, 1.2 equiv.), Fe_3_O_4_@SiO_2_–NH_2_–Pd(ii) (25 mg), CuI (5 mol%), PPh_3_ (10 mol%), Et_3_N (1.5 equiv.), H_2_O (2.5 mL), 90 °C, N_2_ atmosphere. Reaction time as indicated. Isolated yield. TON and TOF calculated based on Pd loading determined by ICP-OES (1.20 wt%).

b Isolated yield.

The turnover number (TON) and turnover frequency (TOF) for each entry were calculated based on the amount of palladium used and are summarized in Table 3.

TON was calculated as the molar ratio of product formed to the amount of palladium present in the catalyst. TOF was determined by dividing TON by the reaction time.

The Pd amount used for TON and TOF calculations was determined from the ICP-OES measured Pd loading (1.20 wt%).

### 2.4 Hot filtration test

To confirm the heterogeneous nature of the Fe_3_O_4_@SiO_2_–NH_2_–Pd(ii) catalyst, a hot filtration test was performed under optimized reaction conditions. In a typical procedure, a mixture of 1a (0.25 mmol), 2a (0.3 mmol), CuI (5 mol%), PPh_3_ (10 mol%), and Et_3_N (1.5 equiv.) in 2.5 mL of water was stirred with Fe_3_O_4_@SiO_2_–NH_2_–Pd(ii) (25 mg) at 90 °C under a nitrogen atmosphere. After 3 minutes of reaction, the magnetic catalyst was rapidly separated from the hot reaction mixture using a strong external magnet. The supernatant was immediately transferred to a preheated reaction vessel and maintained at 90 °C for an additional 1 hour under identical conditions. Upon completion, the reaction mixture was extracted and purified by column chromatography to isolate the product. The isolated yield was determined and compared with the standard reaction. A significant suppression of product formation after catalyst removal would indicate that the catalysis is primarily heterogeneous in nature, with minimal contribution from leached active species.

### 2.5 Gram-scale Sonogashira coupling

To evaluate the scalability of the catalytic system, a gram-scale reaction was performed under the optimized conditions. 1a (5 mmol, 1.06 g) and 2a (6 mmol, 0.61 mL) were reacted in H_2_O (50 mL) containing Fe_3_O_4_@SiO_2_–NH_2_–Pd(ii) (0.50 g, 1.20 wt% Pd), CuI (5 mol%), PPh_3_ (10 mol%), and Et_3_N (7.5 mmol, 1.05 mL). The reaction mixture was stirred at 90 °C under N_2_. After completion, the catalyst was magnetically separated, washed with EtOAc, and dried. The product was purified by column chromatography, affording the coupled product 3a in 99% isolated yield. Notably, the gram-scale reaction exhibited similar kinetics to the small-scale reactions, reaching full conversion within 5 min under identical conditions, indicating that the catalytic system maintains its efficiency upon scale-up.

### 2.6 ICP analysis

The palladium content in the Fe_3_O_4_@SiO_2_–NH_2_–Pd(ii) catalyst was quantitatively analysed using inductively coupled plasma optical emission spectroscopy (ICP-OES, Optima 3000 DV, PerkinElmer). Samples were prepared by digesting an accurately weighed amount of the fresh catalyst and those recovered after 5, 10 and 15 catalytic cycles in concentrated nitric acid under heating until complete dissolution. The resulting solutions were diluted with deionized water to a known volume prior to analysis. The extent of Pd leaching was calculated by comparing the Pd content before and after reuse.

### 2.7 Catalyst characterisation

Comprehensive characterisation of the fresh and recycled catalysts was performed using FT-IR, XRD, TEM, SEM-EDX, VSM, TGA, BET, XPS, and ICP-OES techniques. Detailed experimental procedures and instrumental specifications are provided in the SI (Section S2).

## 3. Results and discussion

### 3.1 Catalyst preparation and characterisation

**Scheme 1:**
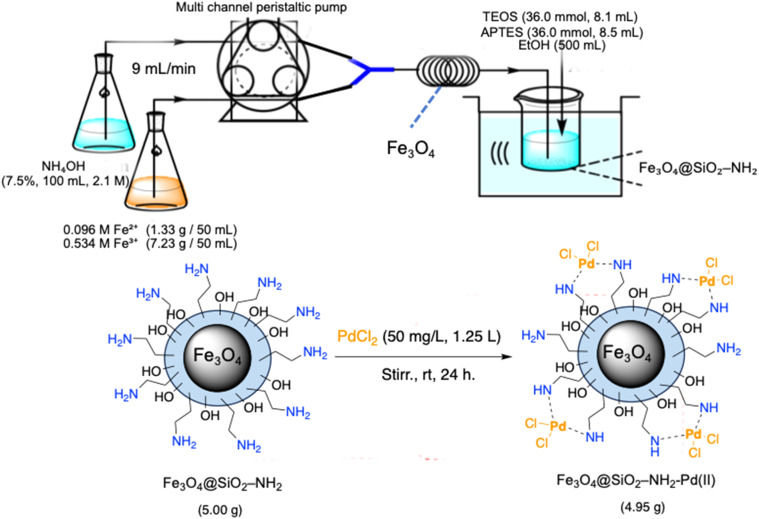
Schematic illustration of the continuous-flow synthesis of Fe_3_O_4_@SiO_2_–NH_2_ nanoparticles and subsequent Pd(ii) immobilisation to yield Fe_3_O_4_@SiO_2_–NH_2_–Pd(ii).

Scheme 1 illustrates a streamlined and scalable synthetic strategy for the preparation of amino-functionalised silica-coated magnetite nanoparticles (Fe_3_O_4_@SiO_2_–NH_2_) and their subsequent coordination with palladium(ii) ions to afford the heterogeneous nanocatalyst Fe_3_O_4_@SiO_2_–NH_2_–Pd(ii). A key advantage of this approach lies in its operational simplicity, scalability, and reliance on readily available reagents under relatively mild conditions.

The magnetite core was synthesised *via* a continuous-flow coprecipitation method previously developed by our group, which enables rapid and reproducible nanoparticle formation without the need for high-temperature reflux or inert atmosphere.^38,39^ In the present work, this method was successfully scaled from gram to decagram quantities, allowing the preparation of more than 10 g of Fe_3_O_4_@SiO_2_–NH_2_ in a single run. This scalability demonstrates the suitability of the process for pilot-scale and potentially industrial applications.

Immediately after formation, the magnetite dispersion was coated with a silica shell and functionalised with (3-aminopropyl)triethoxysilane (APTES) under ultrasonic irradiation in ethanol at 80 °C. This single-pot silanisation and amination step generated a uniform silica layer enriched with surface amine groups, which play a crucial role in stabilising metal ions and preventing aggregation during catalysis. The resulting Fe_3_O_4_@SiO_2_–NH_2_ nanoparticles therefore provide an ideal platform for palladium immobilisation.

Immobilisation of Pd(ii) species was achieved by suspending the Fe_3_O_4_@SiO_2_–NH_2_ support in a dilute aqueous PdCl_2_ solution (50 mg L^−1^) under ambient stirring for 24 h. The surface amine groups strongly coordinate Pd^2+^ ions through chelation, enabling efficient metal anchoring without the need for external ligands or reducing agents. ICP-OES analysis revealed a palladium loading of approximately 1.20 wt%, while SEM-EDX mapping and XPS measurements confirmed the homogeneous distribution and stable chemical environment of Pd species on the support. Transmission electron microscopy (TEM) analysis (Fig. S4) further confirmed the nanoscale morphology of the Fe_3_O_4_@SiO_2_–NH_2_–Pd(ii) catalyst. The nanoparticles exhibit a relatively uniform size distribution with a well-defined core–shell structure consisting of a magnetite core surrounded by a thin silica layer.

To quantitatively evaluate the particle size distribution, the diameters of more than 120 individual nanoparticles were measured from the TEM micrographs. The average particle diameter of the fresh catalyst was determined to be 24.6 ± 2.3 nm, indicating a relatively narrow size distribution. After 15 catalytic cycles, the recycled catalyst showed a comparable average particle size of 25.5 ± 2.7 nm, suggesting that the nanocatalyst maintains its structural integrity during repeated catalytic runs. The minimal change in particle size after recycling indicates that significant nanoparticle aggregation does not occur under the reaction conditions, which is consistent with the observed catalytic stability.

An additional advantage of this catalyst design is its magnetic recoverability. The Fe_3_O_4_ core allows rapid and efficient separation of the catalyst from the reaction medium using an external magnet, eliminating the need for filtration or centrifugation and reducing solvent consumption during work-up. Furthermore, the modular nature of the synthesis allows the palladium content to be tuned by adjusting either the Pd precursor concentration or the surface amine density, offering flexibility for catalyst optimisation.

Overall, the synthetic methodology depicted in Scheme 1 provides a reproducible, energy-efficient, and scalable route to a magnetically recoverable Pd(ii) nanocatalyst. The combination of gram-scale feasibility, strong metal–support interactions, and magnetic separability establishes Fe_3_O_4_@SiO_2_–NH_2_–Pd(ii) as a promising platform for sustainable cross-coupling catalysis.

### 3.2 Optimisation of reaction parameters

#### 3.2.1 Effect of catalyst loading and base (Table 1)

The influence of catalyst loading and base on the aqueous Sonogashira coupling between 2-iodoaniline and phenylacetylene was systematically investigated (Table 1). In the absence of Fe_3_O_4_@SiO_2_–NH_2_–Pd(ii) (entry 1), only 32% yield was obtained after 60 min, which can be attributed to limited background reactivity arising from the copper co-catalyst and base alone.

Introduction of a small amount of catalyst (5 mg, entry 2) resulted in a dramatic enhancement in activity, affording 79% yield within 20 min. Increasing the catalyst loading progressively improved both reaction rate and yield, with an optimal performance observed at 25 mg, delivering 99% isolated yield within only 5 min (entry 6). Further increasing the catalyst amount to 30 mg did not lead to any additional improvement (entry 7), indicating that 25 mg represents the optimal loading under the investigated conditions.

The nature of the base was found to exert a pronounced effect on catalytic performance. Among the bases examined (entries 8–13), triethylamine (Et_3_N) proved to be the most effective, providing near-quantitative yield. In contrast, pyridine and inorganic bases such as KOH, NaOH, K_2_CO_3_, Na_2_CO_3_, and NaOAc afforded lower yields and required longer reaction times. This behaviour can be rationalised by the superior solubility and appropriate basicity of Et_3_N in the aqueous medium, which facilitates efficient deprotonation of the terminal alkyne and promotes smooth catalytic turnover.

#### 3.2.2 Effect of CuI, PPh_3_, and temperature (Table 2)

The roles of the copper co-catalyst, phosphine ligand, and reaction temperature were further examined (Table 2). In the absence of CuI (entry 1), the reaction proceeded very slowly, affording only 38% yield after 60 min, highlighting the essential role of copper in alkyne activation and transmetalation. An optimal CuI loading of 5 mol% was identified (entry 3), delivering 99% yield within 5 min. Both lower and higher CuI amounts resulted in inferior or unchanged performance.

Triphenylphosphine (PPh_3_) was also critical for achieving high activity. Without PPh_3_ (entry 5), the yield dropped sharply to 20%, while increasing the ligand loading up to 10 mol% led to optimal performance (entry 9). A further increase to 12.5 mol% caused a slight decrease in yield, likely due to excessive ligand coordination inhibiting regeneration of the active Pd(0) species.

Temperature played a decisive role in reaction efficiency. At room temperature, the reaction was sluggish (entry 11), whereas moderate heating at 60 °C resulted in incomplete conversion even after extended times (entry 12). The optimal temperature was found to be 90 °C, which provided sufficient activation energy for oxidative addition and reductive elimination steps while maintaining catalyst stability. Collectively, the optimal conditions consisted of 25 mg catalyst, Et_3_N as base, CuI (5 mol%), PPh_3_ (10 mol%), and water as solvent at 90 °C.

### 3.3 Substrate scope and electronic effects

Under the optimized reaction conditions, a series of iodoaniline derivatives were subjected to Sonogashira cross-coupling with various terminal alkynes in water. Aryl iodides were selected as model substrates because oxidative addition of the C–I bond to Pd catalysts generally occurs more readily than that of aryl bromides or chlorides. This higher reactivity enables efficient coupling under the mild aqueous conditions employed in this work (Table 3). Unsubstituted 2-iodoaniline reacted rapidly with various alkynes, affording the corresponding 2-alkynylaniline derivatives in up to 99% isolated yield within 5 min, corresponding to a TOF of 17.12 min^−1^. In comparison, 4-iodoaniline derivatives required slightly longer reaction times and exhibited lower TOF values, which can be attributed to reduced *ortho*-coordination effects that may facilitate oxidative addition in the case of 2-iodoaniline substrates.

Substrates bearing electron-withdrawing groups such as –NO_2_ or –COCH_3_ displayed decreased reaction rates and required extended reaction times (15–60 min), although good to excellent yields (75–98%) were still obtained. These observations indicate that electronic effects play a significant role in governing the reaction kinetics. The Hammett analysis further suggests that substituent effects on the alkyne component are less pronounced than those on the aryl iodide, indicating that electronic variations on the alkyne play a secondary role in the catalytic cycle.

To gain mechanistic insight, Hammett plots were constructed by correlating ln(TOF) values with Hammett *σ* constants for substituents on the aryl iodide (R^1^) and alkyne (R^2^) components. A clear negative linear correlation was observed for R^1^ substituents (*ρ* ≈ −1.1 to −1.2; Fig. S1), indicating that electron-donating groups accelerate the reaction by facilitating oxidative addition of the aryl iodide to Pd(0), which is widely accepted as the rate-determining step in Sonogashira coupling. In contrast, substituent effects on the alkyne were less pronounced (*ρ* ≈ −0.3 to −0.7; Fig. S2), suggesting a secondary influence on steps such as transmetalation or reductive elimination.

### 3.4 Catalyst reusability, palladium retention, and gram-scale application

**Fig. 1:**
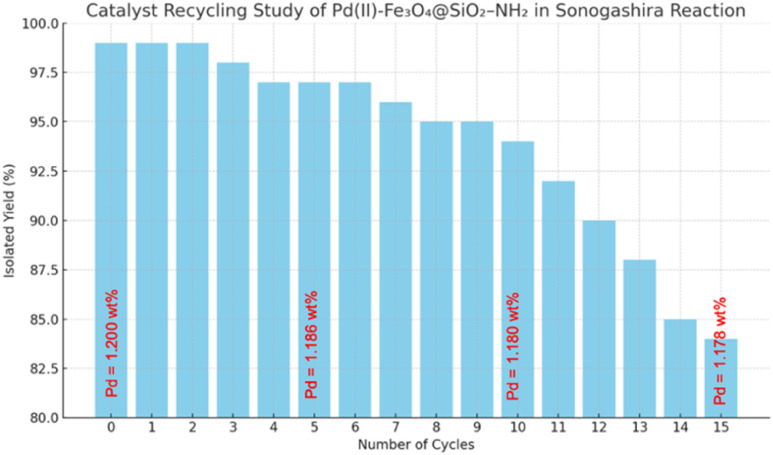
Reusability profile of Fe_3_O_4_@SiO_2_–NH_2_–Pd(ii) catalyst over 15 consecutive cycles in the Sonogashira coupling of 2-iodoaniline and phenylacetylene under optimised aqueous conditions.

The recyclability of Fe_3_O_4_@SiO_2_–NH_2_–Pd(ii) was evaluated over 15 consecutive catalytic cycles using the model reaction between 2-iodoaniline and phenylacetylene (Fig. 1). Near-quantitative yields (98–99%) were maintained over the first five cycles, demonstrating excellent catalyst stability. A gradual decline in activity was observed after prolonged reuse, with yields decreasing to 84% by the 15th cycle.

ICP-OES analysis revealed only a marginal decrease in palladium content from 1.200 wt% in the fresh catalyst to 1.178 wt% after 15 cycles, corresponding to a total Pd loss of approximately 1.83%. These results confirm that palladium leaching is minimal and that the strong Pd–NH_2_ coordination effectively stabilises the metal under aqueous and thermal conditions.

A hot-filtration experiment was performed to examine the heterogeneous nature of the catalyst. Under the optimised conditions, the catalyst was magnetically removed after 3 min of reaction and the filtrate was allowed to react further at 90 °C. Only a marginal increase in product formation was observed, affording 68% yield compared with 99% obtained in the standard reaction containing the catalyst. This pronounced suppression of further conversion indicates that the catalytically active species remain predominantly associated with the solid catalyst. ICP-OES analysis further confirmed minimal palladium leaching during catalysis, with the Pd content decreasing only slightly from 1.200 wt% in the fresh catalyst to 1.178 wt% after 15 cycles. Together with the excellent recyclability observed over fifteen consecutive runs, these results strongly support that the Sonogashira coupling proceeds predominantly through a heterogeneous catalytic pathway, although a minor contribution from soluble Pd species cannot be completely excluded.

The slight loss of activity upon extended reuse is attributed to cumulative effects such as surface fouling, partial Pd reduction or aggregation, minor structural degradation of the aminated silica shell, or physical loss of catalyst during recovery. Importantly, the practical utility of the catalyst was demonstrated through a gram-scale reaction, which afforded the desired coupling product in 99% isolated yield under identical aqueous conditions, highlighting the scalability of the system.

Under the optimised conditions, the model reaction consistently delivered high conversions within short reaction times, demonstrating the sustained intrinsic activity of the catalyst across repeated catalytic cycles. A positional effect was also observed: while 2-iodoaniline substrates reacted more efficiently, 4-iodoaniline analogs required longer reaction times (10 min) and exhibited slightly lower TOFs (8.04–8.13 min^−1^), likely due to reduced *ortho* chelation that may affect Pd coordination and oxidative addition efficiency.

These trends remained consistent throughout repeated catalytic cycles, indicating that the intrinsic positional effects are preserved upon catalyst reuse and reflect the structural robustness of the catalyst.

### 3.5 Structural stability after recycling

Comprehensive post-reaction characterisation confirmed the structural integrity of the catalyst after repeated use. FTIR, TEM, XRD, TGA, SEM-EDX, XPS, BET, and magnetic measurements (Fig. S3–S10) showed only minor changes in surface chemistry, particle size, porosity, and magnetic properties after 15 cycles. These results collectively demonstrate that the Fe_3_O_4_@SiO_2_–NH_2_–Pd(ii) catalyst maintains its core–shell architecture, mesoporosity, and magnetic recoverability, consistent with its sustained catalytic performance.

### 3.6 Comparison with reported catalysts

A comparison with representative literature-reported Sonogashira catalysts (Table 4) highlights the advantages of the present system. Fe_3_O_4_@SiO_2_–NH_2_–Pd(ii) combines high activity (99% yield in 5 min), high TOF (17.12 min^−1^), aqueous reaction conditions, and excellent recyclability. In contrast, many reported systems require longer reaction times, higher temperatures, or organic solvents and exhibit significantly lower TOF values. The superior performance of the present catalyst can be attributed to the synergistic effects of the magnetic core–shell structure, amino-functionalised silica interface, and well-dispersed Pd(ii) species.

**Table 4:** Comparison with representative Sonogashira catalytic systems reported in the literature

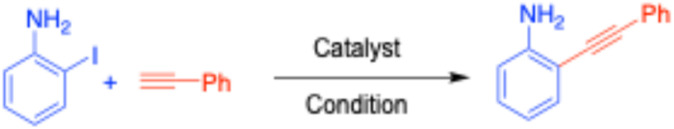						
Catalyst [ref.]	Condition	Yield (%)	Time (min)	TON	TOF (min^−1^)	Reuse
Fe_3_O_4_@SiO_2_–NH_2_–Pd(ii) (this work)	CuI, PPh_3_, Et_3_N, H_2_O, 90 °C	99	5	85.6	17.12	≥15 cycles
Pd(0,ii)/CuO–Fe_3_O_4_ (ref. 30)	NaOH, PhMe, 130 °C	68	4320	295.6	6.8 × 10^−2^	—
CuI/DABCO^40^	Cs_2_CO_3_, DMF, 135–140 °C	89	480	4.5	9.27 × 10^−3^	—
(PPh_3_)_2_CuBH_4_ (ref. 41)	DBU, EtOH, 120 °C	96	1440	19.2	1.33 × 10^−2^	—
Cu_2_I_2_-((−)-sparteine)_2_ complex^42^	Cs_2_CO_3_, DMF, 120 °C	92	480	18.4	3.83 × 10^−2^	—

### 3.7 Proposed catalytic mechanism and role of the magnetic nanocatalyst

**Scheme 2:**
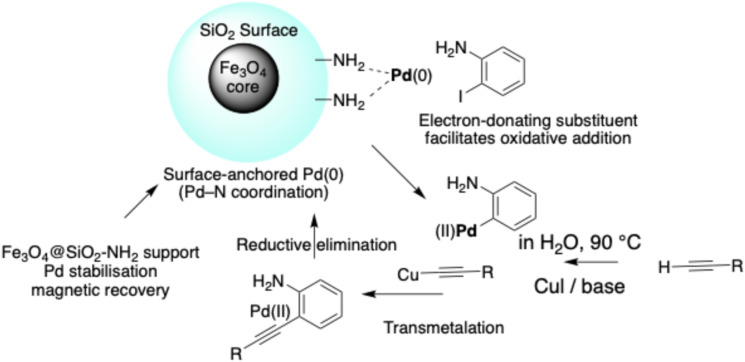
Proposed heterogeneous Pd(0)/Pd(ii) catalytic cycle for aqueous Sonogashira coupling over surface-anchored Fe_3_O_4_@SiO_2_–NH_2_–Pd nanocatalyst. The catalyst structure (left inset; see Scheme 1 for synthesis) illustrates Pd–N coordination sites responsible for stabilising active Pd species during heterogeneous catalysis. Electron donation from amino-substituted aryl iodides is proposed to facilitate oxidative addition, which is likely involved in the rate-determining step.

The Sonogashira coupling catalysed by Fe_3_O_4_@SiO_2_–NH_2_–Pd(ii) is proposed to proceed through a heterogeneous Pd(0)/Pd(ii) catalytic cycle, as illustrated in Scheme 2. Under the reaction conditions, Pd(ii) species coordinated to the amino-functionalised silica surface are partially reduced *in situ* to generate catalytically active Pd(0) centres, which function as the primary active sites for cross-coupling.

The catalytic cycle is initiated by oxidative addition of the aryl iodide to surface-bound Pd(0), affording an aryl–Pd(ii) intermediate. Simultaneously, the terminal alkyne undergoes copper-assisted activation to form a copper acetylide species. Subsequent transmetalation between the aryl–Pd(ii) intermediate and the activated alkyne generates a Pd(ii) alkynyl complex, which undergoes reductive elimination to produce the desired C–C coupled product while regenerating the Pd(0) active species.

In contrast to homogeneous systems, the amino-functionalised silica layer plays a crucial stabilising role by coordinating palladium species through Pd–N interactions, thereby suppressing aggregation and minimising metal leaching during catalysis. Although the Fe_3_O_4_ magnetic core does not directly participate in the electronic steps of the catalytic cycle, it enables rapid magnetic separation and efficient catalyst recovery, contributing to preservation of active-site integrity over repeated cycles.

Mechanistic insight is further supported by the observed Hammett correlation, which indicates that electron-donating substituents on the aryl iodide accelerate the reaction rate. This trend suggests that oxidative addition is likely involved in, or closely associated with, the rate-determining step, consistent with typical heterogeneous Pd-catalysed Sonogashira mechanisms reported in the literature. The stabilising coordination environment provided by the amino-functionalised support is therefore proposed to facilitate efficient oxidative addition while maintaining dispersion of catalytically active Pd species. Hot-filtration experiments and ICP-OES analysis further confirm the predominantly heterogeneous nature of the catalytic process, showing negligible palladium leaching. These results suggest that the catalytic process is predominantly heterogeneous, although a minor contribution from soluble Pd species cannot be completely excluded.

## 4. Conclusions

In summary, a magnetically recoverable Pd(ii)-anchored nanocatalyst based on amino-functionalised silica-coated magnetite (Fe_3_O_4_@SiO_2_–NH_2_–Pd(ii)) has been developed *via* a scalable continuous-flow synthesis and successfully applied to Sonogashira cross-coupling reactions conducted in water. The catalyst enables efficient C–C bond formation between iodoanilines and terminal alkynes, delivering high isolated yields under mild aqueous conditions while allowing rapid magnetic separation and reuse. Recyclability studies over 15 consecutive cycles, together with hot filtration experiments and ICP-OES analysis, indicate that the catalytic process proceeds predominantly *via* a heterogeneous pathway with only minimal palladium leaching. Hammett analysis reveals clear electronic effects consistent with oxidative addition playing a key role in the catalytic cycle. The integration of continuous-flow nanomaterial synthesis, aqueous-phase catalysis, and magnetic recyclability underscores the practical potential of this system for more sustainable palladium-catalysed cross-coupling processes.

## Conflicts of interest

There are no conflicts to declare.

## Supplementary Material

RA-016-D6RA01007E-s001

## Data Availability

The data supporting this article are available within the article and its supplementary information (SI). Supplementary information: detailed experimental procedures, full characterisation data (FT-IR, XRD, TEM, SEM-EDX, BET, VSM, and ICP-OES), additional catalytic studies, optimisation tables, recycling experiments, and mechanistic investigations including Hammett analysis and kinetic data; spectroscopic data (^1^H and ^13^C NMR, and HRMS) for all synthesised compounds See DOI: https://doi.org/10.1039/d6ra01007e.
