# Supplementary material for: Continuous-flow synthesis of Pd(ii)-anchored amino-functionalised magnetic silica nanoparticles as a robust recyclable catalyst for aqueous Sonogashira cross-coupling
Source: RSC Adv. 2026 Mar 23;16(18):16145–55. doi: 10.1039/d6ra01007e (PMC13007890; doi:10.1039/d6ra01007e)
Supplement: RA-016-D6RA01007E-s001 [file RA-016-D6RA01007E-s001.pdf]

Supplementary Information (SI) for RSC Advances.
This journal is © The Royal Society of Chemistry 2026

Supporting Information (SI)

Continuous-flow synthesis of Pd(II)-anchored amino-functionalised magnetic silica nanoparticles as a robust recyclable catalyst for aqueous Sonogashira cross-coupling

Uthai Sakee,* Aekkaphon Mokkarat, Widchaya Radchatawedchakoon, Senee Kruanetr, and Ratchaneekorn Pilasombat

Creative Chemistry and Innovation Research Unit,Center of Excellence for Innovation in Chemistry (PERCH-CIC), Department of Chemistry, Faculty of Science, Mahasarakham University, Mahasarakham 44150, Thailand

Corresponding author: uthai.s@msu.ac.th

Table of Contents

S1. General Information

S2. Catalyst Characterisation

S3. Textural Properties

S4. Catalytic Studies and Hammett Analysis

S5. Catalyst Characterisation Before and After Recycling

S6. Characterisation Data of Products (3a–3t)

S7. ^{1}H NMR Spectra of Products

S8. ^{13}C NMR Spectra of Products

S9. HRMS spectra of selected products.

S10. Literature References for Known Compounds

S1. General Information

All reagents and solvents were purchased from commercial suppliers (Sigma-Aldrich, Merck, TCI, and Acros Organics) and were used as received without further purification unless otherwise noted.

^{1}H NMR spectra were recorded on a Varian Mercury Plus 400 MHz spectrometer using $CDCl_3$ as solvent with tetramethylsilane (TMS) as internal standard. Chemical shifts (δ) are reported in ppm and coupling constants (J) in Hz.

Multiplicity abbreviations are as follows:

s = singlet; d = doublet; t = triplet; q = quartet; dd = doublet of doublets; dt = doublet of triplets; m = multiplet; br = broad.

High-resolution mass spectra (HRMS) were recorded on a Bruker micrOTOF-Q II mass spectrometer equipped with an electrospray ionisation (ESI) source operating in positive-ion mode.

Thin-layer chromatography (TLC) was performed on silica gel 60 F254 plates and visualised under UV light.

S2. Catalyst Characterisation

Comprehensive physicochemical analyses were carried out to evaluate the structural, surface, magnetic, and thermal properties of the synthesised catalysts described in Section 2.2 of the main manuscript.

FT-IR spectroscopy: FT-IR spectra were recorded on a Bruker INVENIO-S spectrometer in the range 4000–400 cm^{-1}.

TEM images were obtained using a JEOL JEM-1230 transmission electron microscope operating at 200 kV.

X-ray Diffraction (XRD): XRD patterns were recorded using a Bruker D8 Advance diffractometer with Cu Kα radiation (λ = 1.5406 Å).

Vibrating Sample Magnetometry (VSM): Magnetic measurements were performed at room temperature using a Lakeshore VSM system.

Thermogravimetric Analysis (TGA): TGA measurements were conducted under nitrogen atmosphere at a heating rate of 10 °C min^{-1}.

N_2 Adsorption–Desorption (BET): Surface area and porosity were determined using a Micromeritics ASAP 2020 instrument. Samples were degassed prior to measurement.

ICP–OES: Pd loading and metal leaching were quantified using ICP–OES after acid digestion of catalyst samples. These measurements support the heterogeneous nature of the catalytic system discussed in Section 3.7 of the main manuscript.

S3. Textural Properties

BET Surface Area Analysis

Surface areas were calculated using the Brunauer–Emmett–Teller (BET) method (P/P_0 = 0.05–0.30).

Pore size distributions were obtained from the desorption branch using the Barrett–Joyner–Halenda (BJH) method based on the Kelvin equation.

Table S1. Textural properties of Fe_3O_4@SiO_2–NH_2–Pd(II) nanocatalyst (a) before and (b) after 15 catalytic cycles as determined by BET and BJH analysis.[a]

Sample	Surface area (m^2 g^{-1})	Pore volume (cm^3 g^{-1})	Pore size (nm)
a) Fe_3O_4@SiO_2-NH_2 -Pd(II)	206.78	0.51	7.23

b) Fe_3O_4@SiO_2-NH_2 -Pd(II)	199.12	0.48	7.04

[a] Values were calculated by the BET method; pore size distribution was obtained using the BJH method.

S4. Catalytic Studies and Hammett Analysis

General Procedure

Initial rates were determined under identical reaction conditions using substituted aryl iodides. Conversions below 15% were used to calculate initial rates.

TOF values were calculated using:

$$TOF = (\text{mol product})/(\text{mol Pd} \times \text{time})$$

ln(TOF) values were plotted against Hammett σ constants obtained from literature.

Detailed kinetic data and Hammett plots used to evaluate electronic substituent effects in aqueous Sonogashira coupling are provided in Figures S1–S2.

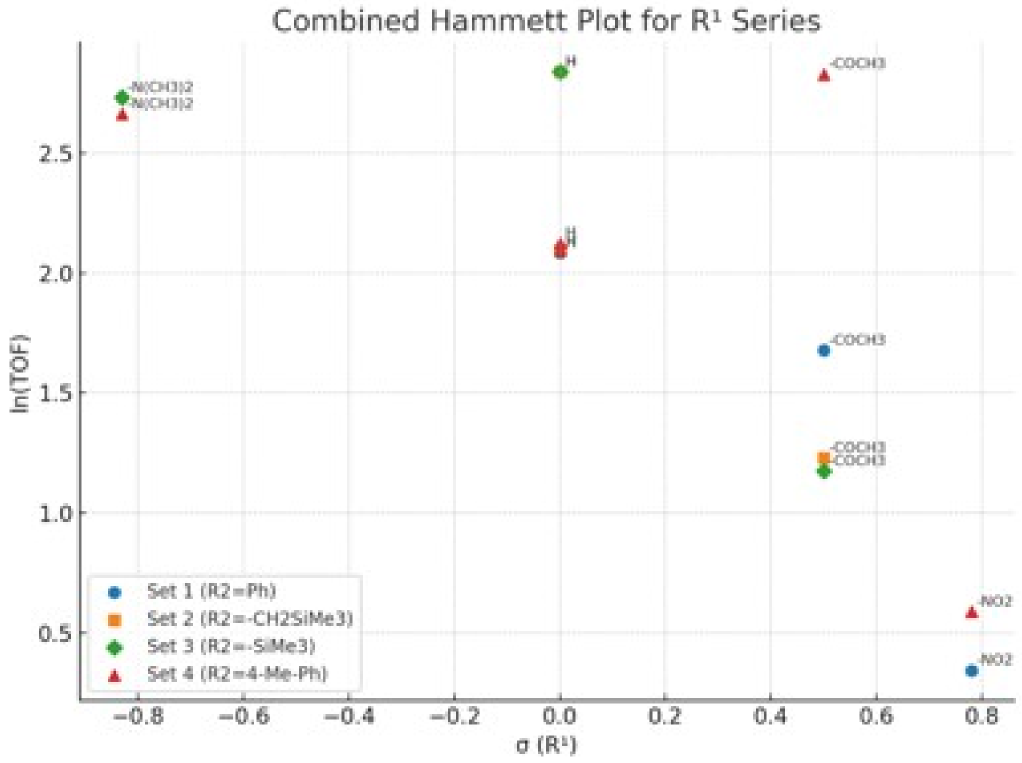

Figure S1. The correlation between the Hammett sigma constants (σ) of R^1 substituents and the natural logarithm of TOF (ln(TOF)) for different R^2 groups: Ph (●), CH_2SiMe_3 (■), $SiMe_3$ (◆), and 4-Me-Ph (▲). The electronic effects of R^1 significantly influence catalytic activity, particularly for electron-donating ($–NMe_2$) and electron-withdrawing ($–NO_2$, $–COCH_3$) groups

.

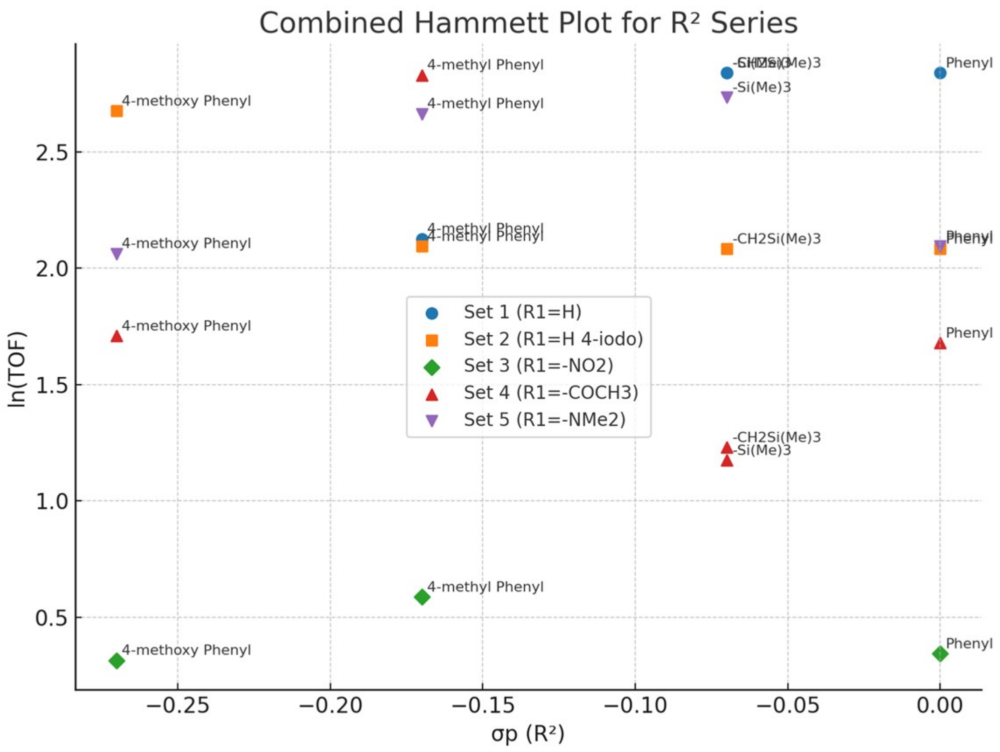

Figure S2. The correlation between σ_p (Hammett constants) of various R^2 substituents and ln(TOF) for five representative R^1 groups: H (●), H–4-Iodo (■), NO_2 (◆), $COCH_3$ (▲), and NMe_2 (▼). The results suggest that R^2 groups also influence catalytic activity, although the effect is less pronounced than that of R^1 substituents.

S5. Catalyst Characterisation Before and After Recycling

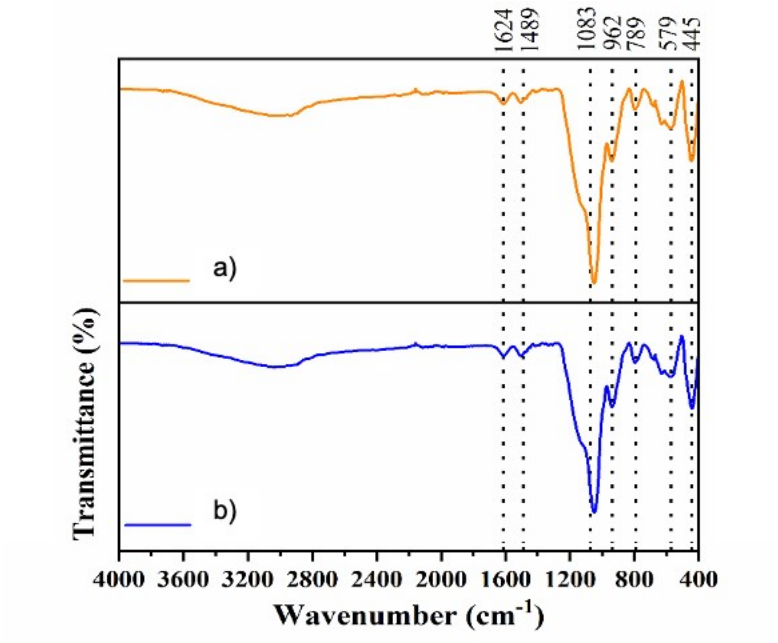

Figure S3. FTIR spectra of Fe_3O_4@SiO_2–NH_2–Pd(II) nanocatalyst before (b) and after (a) 15 catalytic cycles, demonstrating the retention of key functional groups and structural stability.

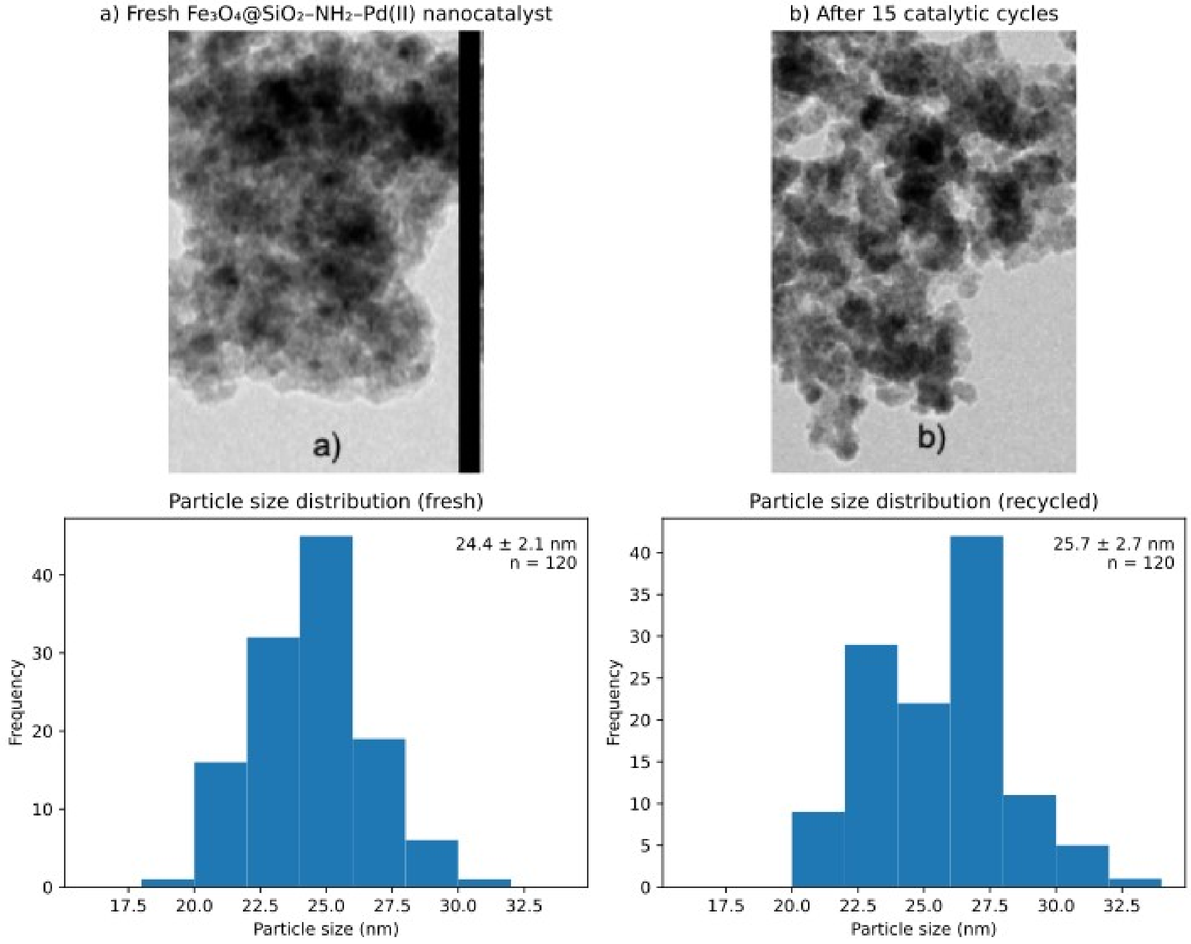

Figure S4. TEM images of Fe_3O_4@SiO_2–NH_2–Pd(II) nanocatalyst (a) before catalysis and (b) after 15 catalytic cycles showing the corresponding particle size distributions, together with the corresponding particle size distributions determined from measurements of more than 120 nanoparticles in the TEM images. The average particle sizes were determined to be 24.6 ± 2.3 nm for the fresh catalyst and 25.5 ± 2.7 nm after catalytic recycling.

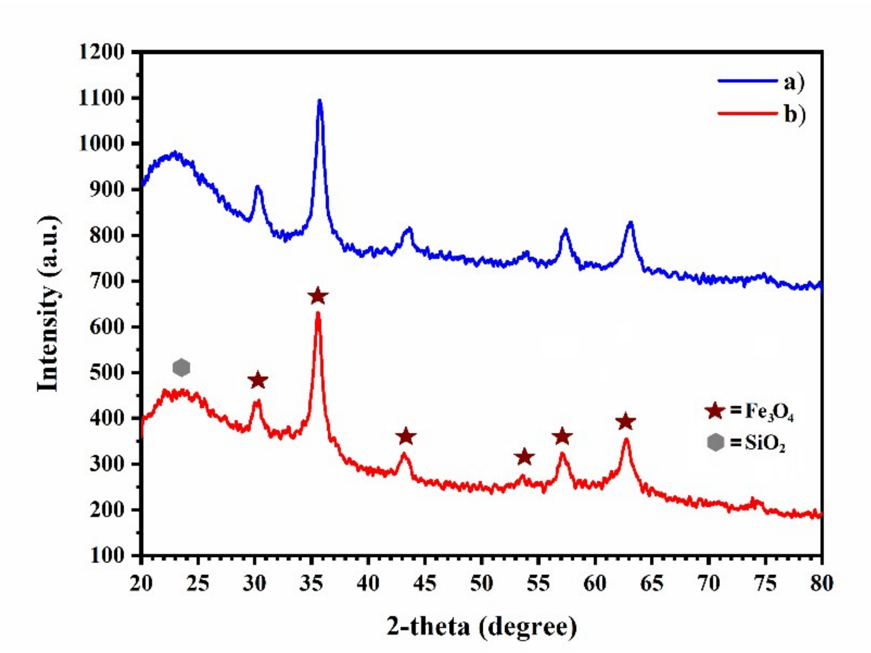

Figure S5. Powder XRD patterns of $Fe_3O_4@SiO_2–NH_2–Pd(II)$ nanocatalyst before (a) and after (b) 15 catalytic cycles, confirming crystallographic stability of the magnetite core and silica shell.

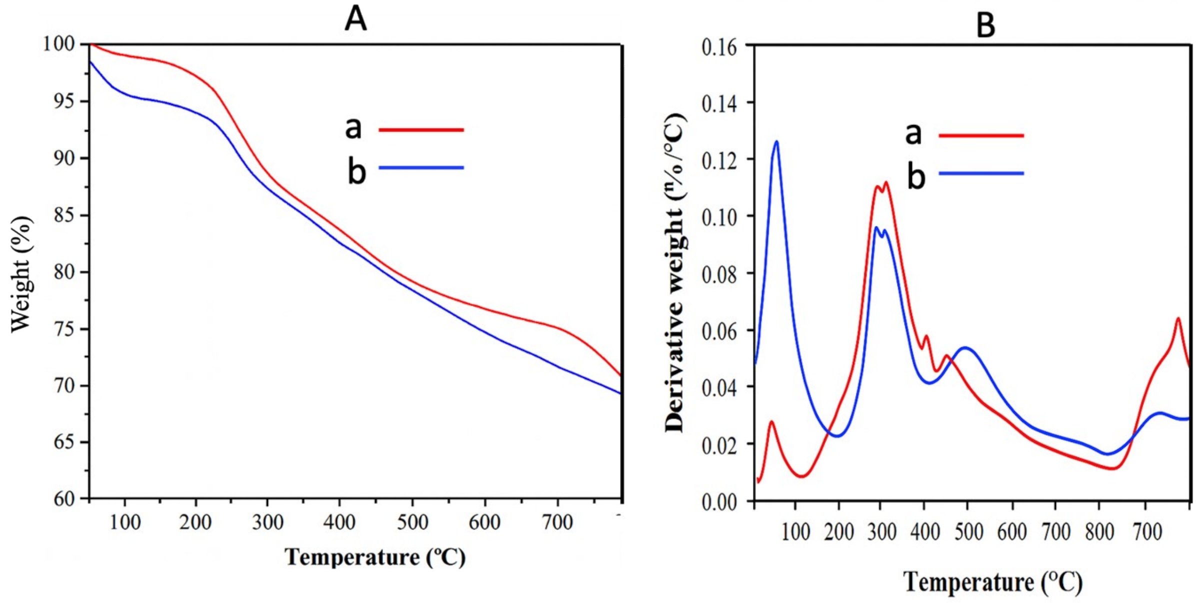

Figure S6. (A) TGA and (B) DTG curves of $Fe_3O_4@SiO_2–NH_2–Pd(II)$ nanocatalyst before (a) and after (b) 15 catalytic cycles, indicating thermal stability and degradation patterns of surface organic moieties.

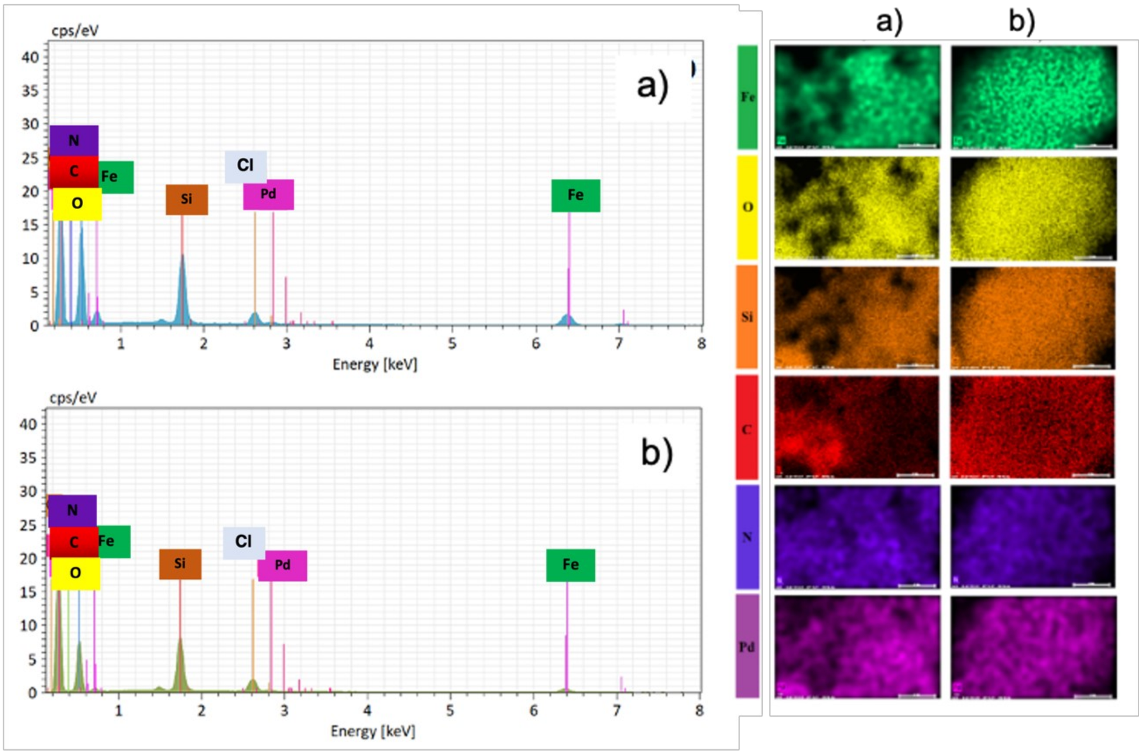

Figure S7. SEM–EDX spectra and corresponding elemental mapping images of

(a) fresh Fe_3O_4@SiO_2–NH_2–Pd(II) catalyst and

(b) catalyst recovered after Pd leaching test.

Consistent color coding was used for both spectra and elemental mapping

(Fe = green, O = yellow, Si = orange, C = red, N = purple, Pd = pink).

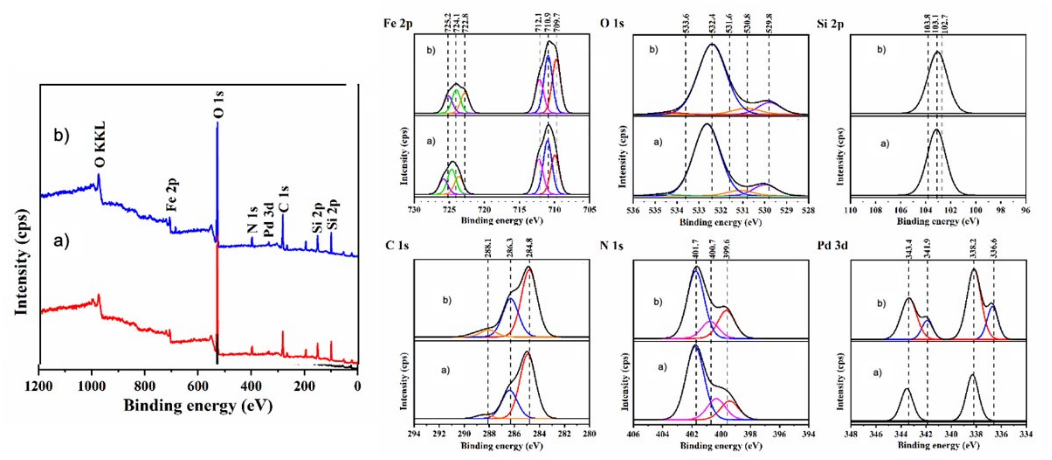

Figure S8. XPS survey and high-resolution spectra of Fe 2p, O 1s, Si 2p, C 1s, N 1s, and Pd 3d regions of $Fe_3O_4@SiO_2–NH_2–Pd(II)$ nanocatalyst before (a) and after (b) 15 catalytic cycles, confirming chemical state preservation.

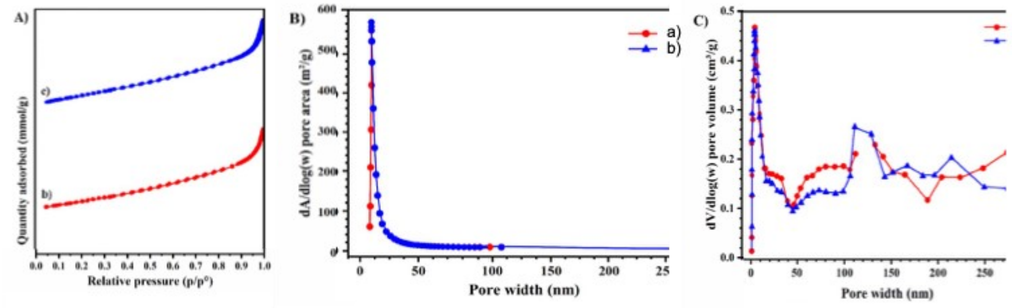

Figure S9. (A) N_2 adsorption–desorption isotherms, (B) BJH pore size distribution, and (C) pore volume profiles of $Fe_3O_4@SiO_2–NH_2–Pd(II)$ nanocatalyst before (a) and after (b) 15 catalytic cycles, highlighting mesoporosity and minimal loss of surface area

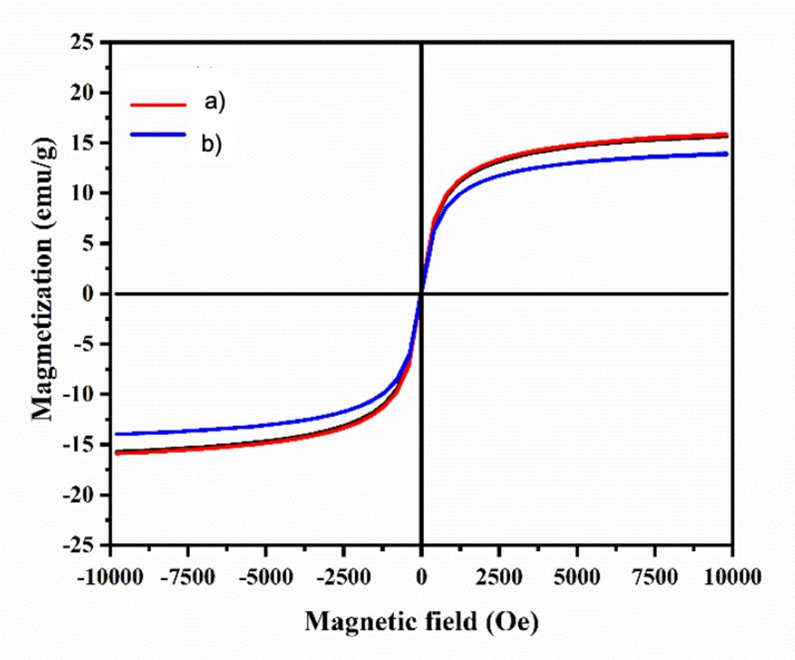

Figure S10. VSM magnetisation curves of Fe_3O_4@SiO_2–NH_2–Pd(II) before (a) and after (b) 15 catalytic cycles.

Table S2. Magnetic parameters of Fe_3O_4@SiO_2–NH_2–Pd(II) nanocatalyst (a) before and (b) after 15 catalytic cycles from VSM analysis.

Sample	M_s (emu g^{-1})	M_r (emu g^{-1})	H_c (Oe)
a) Fe_3O_4@SiO_2-NH_2-Pd(II)	15.60	0.14	7.46
b) Fe_3O_4@SiO_2-NH_2-Pd(II)	14.07	0.14	8.50

S6. Characterisation Data of Products (3a–3t)

2-(Phenylethynyl)aniline (3a)

^{1}H NMR (400 MHz, $CDCl_3$) δ 7.67–7.44 (m, 2H), 7.44–7.19 (m, 4H), 7.19–7.03 (m, 1H), 6.93–6.63 (m, 2H), 5.00 (br s, 2H, NH_2).

^{13}C NMR (100 MHz, $CDCl_3$) δ 147.8, 132.3, 131.6, 129.9, 128.5, 128.4, 123.5, 118.3, 114.6, 108.2, 94.9, 86.0.

2-(3-(Trimethylsilyl)prop-1-yn-1-yl)aniline (3b)

^{1}H NMR (400 MHz, $CDCl_3$) δ 7.27 (d, *J* = 8.0 Hz, 1H), 6.89 (t, *J* = 8.0 Hz, 1H), 6.72–6.67 (m, 2H), 4.08 (br s, 2H, NH_2), 1.81 (s, 2H, CH_2), 0.22 (s, 9H, $Si(CH_3)_3$).
^{13}C NMR (100 MHz, $CDCl_3$) δ 147.6, 132.1, 128.5, 118.0, 114.2, 110.0, 93.8, 76.0, 8.4, -1.8.
HRMS (ESI-TOF) m/z: $[M+H]^+$ calcd for $C_{12}H_{18}NSi$ 204.1203; found 204.1222.

2-((Trimethylsilyl)ethynyl)aniline (3c)

^{1}H NMR (400 MHz, $CDCl_3$) δ 7.29–7.12 (m, 2H), 6.69–6.64 (m, 2H), 4.23 (br s, 2H, NH_2), 0.27 (s, 9H, $Si(CH_3)_3$).
^{13}C NMR (100 MHz, $CDCl_3$) δ 148.4, 132.4, 130.0, 117.1, 114.3, 107.9, 102.0, 99.9, −0.3.

2-(p-Tolylethynyl)aniline (3d)

^{1}H NMR (400 MHz, $CDCl_3$) δ 7.47 (d, *J* = 8.0 Hz, 1H), 7.41 (d, *J* = 8.0 Hz, 1H), 7.21–7.15 (m, 3H), 6.78–6.74 (m, 2H), 4.31 (br s, 2H, NH_2), 2.41 (s, 3H, CH_3).
^{13}C NMR (100 MHz, $CDCl_3$) δ 147.7, 138.3, 132.0, 131.3, 129.5, 129.1, 120.2, 118.0, 114.3, 108.2, 94.8, 85.1, 21.5.

4-(Phenylethynyl)aniline (3e)

^{1}H NMR (400 MHz, $CDCl_3$) δ 7.42–7.20 (m, 4H), 6.55–6.53 (m, 2H), 3.72 (br s, 2H, NH_2).
^{13}C NMR (100 MHz, $CDCl_3$) δ 146.8, 133.1, 131.5, 128.4, 127.8, 124.1, 114.9, 111.8, 90.3, 87.5.

4-(3-(Trimethylsilyl)prop-1-yn-1-yl)aniline (3f)

^{1}H NMR (400 MHz, $CDCl_3$) δ 7.18 (d, *J* = 8.0 Hz, 2H), 6.57 (d, *J* = 8.0 Hz, 2H), 3.07 (br s, 2H, NH_2), 1.67 (s, 2H, CH_2), 0.15 (s, 9H, $Si(CH_3)_3$).
^{13}C NMR (100 MHz, $CDCl_3$) δ 145.6, 132.8, 130.7, 115.9, 114.0, 85.7, 79.9, 8.0, –1.8.
HRMS (ESI-TOF) m/z: $[M+H]^+$ calcd for $C_{12}H_{18}NSi$ 204.1203; found 204.1237.

4-((4-Methoxyphenyl)ethynyl)aniline (3g)

^{1}H NMR (400 MHz, $CDCl_3$) δ 7.24 (d, *J* = 8.0 Hz, 2H), 7.10 (d, *J* = 8.0 Hz, 2H), 6.68 (d, *J* = 8.0 Hz, 2H), 6.45 (d, *J* = 8.0 Hz, 2H), 4.08 (br s, 2H, NH_2), 3.82 (s, 3H, OCH_3).

4-(p-Tolylethynyl)aniline (3h)

^{1}H NMR (400 MHz, $CDCl_3$) δ 7.27 (d, *J* = 8.0 Hz, 2H), 7.01 (d, *J* = 8.0 Hz, 2H), 6.51 (d, *J* = 8.0 Hz, 2H), 3.68 (br s, 2H, NH_2), 2.23 (s, 3H, CH_3).
^{13}C NMR (100 MHz, $CDCl_3$) δ 146.4, 137.7, 132.9, 131.2, 129.0, 120.8, 114.7, 112.9, 89.3, 87.4, 21.4.

4-Nitro-2-(phenylethynyl)aniline (3i)

^{1}H NMR (400 MHz, $CDCl_3$) δ 8.10 (d, *J* = 4.0 Hz, 1H), 7.85 (dd, *J* = 8.0, 1.5 Hz, 1H), 7.36–7.17 (m, 5H), 6.51 (d, *J* = 8.0 Hz, 1H), 4.81 (br s, 2H, NH_2).
^{13}C NMR (100 MHz, $CDCl_3$) δ 152.8, 138.6, 131.6, 129.0, 128.7, 128.5, 125.9, 122.0, 112.9, 107.2, 96.2, 83.3.

2-((4-Methoxyphenyl)ethynyl)-4-nitroaniline (3j)

^{1}H NMR (400 MHz, $CDCl_3$) δ 8.20 (d, *J* = 4.0 Hz, 1H), 7.95 (dd, *J* = 8.0, 1.5 Hz, 1H), 7.41–7.39 (m, 2H), 6.84–6.82 (m, 2H), 6.03 (d, *J* = 8.0 Hz, 1H), 4.95 (br s, 2H, NH_2), 3.77 (s, 3H, OCH_3).
^{13}C NMR (100 MHz, $CDCl_3$) δ 160.1, 152.7, 138.5, 133.1, 128.5, 125.6, 114.2, 112.7, 107.6, 96.3, 82.0, 55.3.

4-Nitro-2-(p-tolylethynyl)aniline (3k)

^{1}H NMR (400 MHz, $CDCl_3$) δ 8.28 (d, *J* = 4.0 Hz, 1H), 8.05–8.00 (m, 1H), 7.43 (d, *J* = 8.0 Hz, 2H), 7.19 (d, *J* = 8.0 Hz, 2H), 6.70 (d, *J* = 8.0 Hz, 1H), 5.02 (br s, 2H, NH_2), 2.38 (s, 3H, CH_3).

^{13}C NMR (100 MHz, $CDCl_3$) δ 152.8, 139.3, 138.5, 135.5, 131.5, 129.3, 129.0, 125.8, 119.0, 112.8, 112.2, 107.4, 96.4, 82.6, 21.5.

1-(4-Amino-3-(phenylethynyl)phenyl)ethan-1-one (3l)

^{1}H NMR (400 MHz, $CDCl_3$) δ 8.01 (s, 1H), 7.79 (dd, *J* = 8.0, 1.5 Hz, 1H), 7.54–7.52 (m, 2H), 7.37–7.35 (m, 3H), 6.71 (d, *J* = 8.0 Hz, 1H), 4.82 (br s, 2H, NH_2), 2.52 (s, 3H, $COCH_3$).

1-(4-Amino-3-(3-(trimethylsilyl)prop-1-yn-1-yl)phenyl)ethan-1-one (3m)

^{1}H NMR (400 MHz, $CDCl_3$) δ 7.79 (d, *J* = 4.0 Hz, 1H), 7.65 (dd, *J* = 8.0, 1.5 Hz, 1H), 6.59 (d, *J* = 8.0 Hz, 1H), 4.54 (br s, 2H, NH_2), 2.42 (s, 3H, $COCH_3$), 1.71 (s, 2H, CH_2), 0.11 (s, 9H, $Si(CH_3)_3$).
HRMS (ESI-TOF) m/z: $[M+H]^+$ calcd for $C_{14}H_{20}NOSi$, 246.1309; found 246.1332.

1-(4-Amino-3-((trimethylsilyl)ethynyl)phenyl)ethan-1-one (3n)

^{1}H NMR (400 MHz, $CDCl_3$) δ 7.92 (s, 1H), 7.75 (dd, *J* = 8.0, 1.5 Hz, 1H), 6.67 (d, *J* = 8.0 Hz, 1H), 4.75 (br s, 2H, NH_2), 2.49 (s, 3H, $COCH_3$), 0.26 (s, 9H, $Si(CH_3)_3$).
^{13}C NMR (100 MHz, $CDCl_3$) δ 196.0, 152.3, 134.1, 130.7, 127.4, 113.4, 106.9, 100.8, 100.7, 26.2, –0.2.

1-(4-Amino-3-((4-methoxyphenyl)ethynyl)phenyl)ethan-1-one (3o)

^{1}H NMR (400 MHz, $CDCl_3$) δ 7.90 (s, 1H), 7.77 (dd, *J* = 8.0, 1.5 Hz, 1H), 7.46 (d, *J* = 8.0 Hz, 2H), 6.88 (d, *J* = 8.0 Hz, 2H), 6.70 (d, *J* = 8.0 Hz, 1H), 4.75 (br s, 2H, NH_2), 3.82 (s, 3H, OCH_3), 2.51 (s, 3H, $COCH_3$).
^{13}C NMR (100 MHz, $CDCl_3$) δ 196.0, 159.8, 151.6, 133.5, 133.0, 130.1, 127.3, 114.8, 114.0, 113.2, 107.2, 95.1, 83.3, 55.3, 26.0.
HRMS (ESI-TOF) m/z: $[M+Na]^+$ calcd for $C_{17}H_{15}NNaO_2$, 288.0995; found 288.1021.

1-(4-Amino-3-(*p*-tolylethynyl)phenyl)ethan-1-one (3p)

^{1}H NMR (400 MHz, $CDCl_3$) δ 7.78 (d, *J* = 4.0 Hz, 1H), 7.56 (dd, *J* = 8.0, 1.5 Hz, 1H), 7.21 (d, *J* = 8.0 Hz, 2H), 6.95 (d, *J* = 8.0 Hz, 2H), 6.49 (d, *J* = 8.0 Hz, 1H), 4.61 (br s, 2H, NH_2), 2.29 (s, 3H, Ar-CH_3), 2.15 (s, 3H, $COCH_3$).

^{13}C NMR (100 MHz, $CDCl_3$) δ 196.0, 151.7, 140.2, 138.7, 133.6, 131.3, 130.2, 129.2, 127.2, 119.6, 113.2, 113.0, 107.0, 95.3, 84.0, 26.0, 21.4.

HRMS (ESI-TOF) m/z: $[M+H]^+$ calcd for $C_{17}H_{17}NO$, 250.1226; found 250.1260.

***N,N*-Dimethyl-4-(phenylethynyl)aniline (3q)**

^{1}H NMR (400 MHz, $CDCl_3$) δ 7.54 (d, *J* = 8.0 Hz, 2H), 7.44 (dd, *J* = 8.0, 1.5 Hz, 2H), 7.36–7.29 (m, 3H), 6.72 (d, *J* = 8.0 Hz, 2H), 3.00 (s, 6H, $N(CH_3)_2$).

^{13}C NMR (100 MHz, $CDCl_3$) δ 150.2, 132.9, 131.5, 128.4, 127.6, 124.3, 112.0, 90.8, 87.5, 40.4.

***N,N*-Dimethyl-4-((trimethylsilyl)ethynyl)aniline (3r)**

^{1}H NMR (400 MHz, $CDCl_3$) δ 7.41 (d, *J* = 8.0 Hz, 2H), 6.43 (d, *J* = 8.0 Hz, 2H), 2.85 (s, 6H, $N(CH_3)_2$), 0.00 (s, 9H, $Si(CH_3)_3$).

4-((4-Methoxyphenyl)ethynyl)-*N,N*-dimethylaniline (3s)

^{1}H NMR (400 MHz, $CDCl_3$) δ 7.44 (d, *J* = 8.0 Hz, 2H), 7.40 (d, *J* = 8.0 Hz, 2H), 6.86 (d, *J* = 8.0 Hz, 2H), 6.67 (d, *J* = 8.0 Hz, 2H), 3.82 (s, 3H, OCH_3), 2.98 (s, 6H, $N(CH_3)_2$).

^{13}C NMR (100 MHz, $CDCl_3$) δ 159.2, 150.1, 134.2, 132.9, 132.7, 116.5, 114.1, 112.1, 89.2, 87.3, 55.4, 40.4.

***N,N*-Dimethyl-4-(p-tolylethynyl)aniline (3t)**

^{1}H NMR (400 MHz, $CDCl_3$) δ 7.21–7.18 (m, 4H), 6.94 (d, *J* = 8.0 Hz, 2H), 6.47 (d, *J* = 8.0 Hz, 2H), 2.78 (s, 6H, $N(CH_3)_2$), 2.31 (s, 3H, Ar-CH_3).

^{13}C NMR (100 MHz, $CDCl_3$) δ 150.0, 137.4, 132.6, 131.1, 129.0, 121.0, 111.8, 110.3, 89.8, 87.4, 40.2, 21.4.

S7. ¹H NMR Spectra of Products

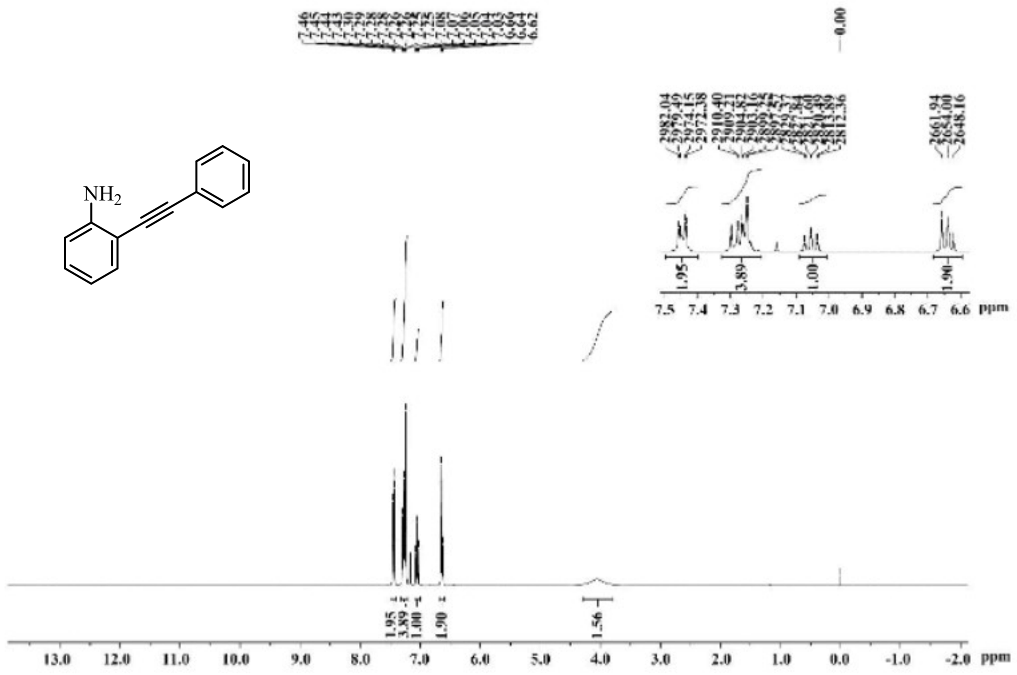

Figure S11. ¹H NMR spectrum of 3a

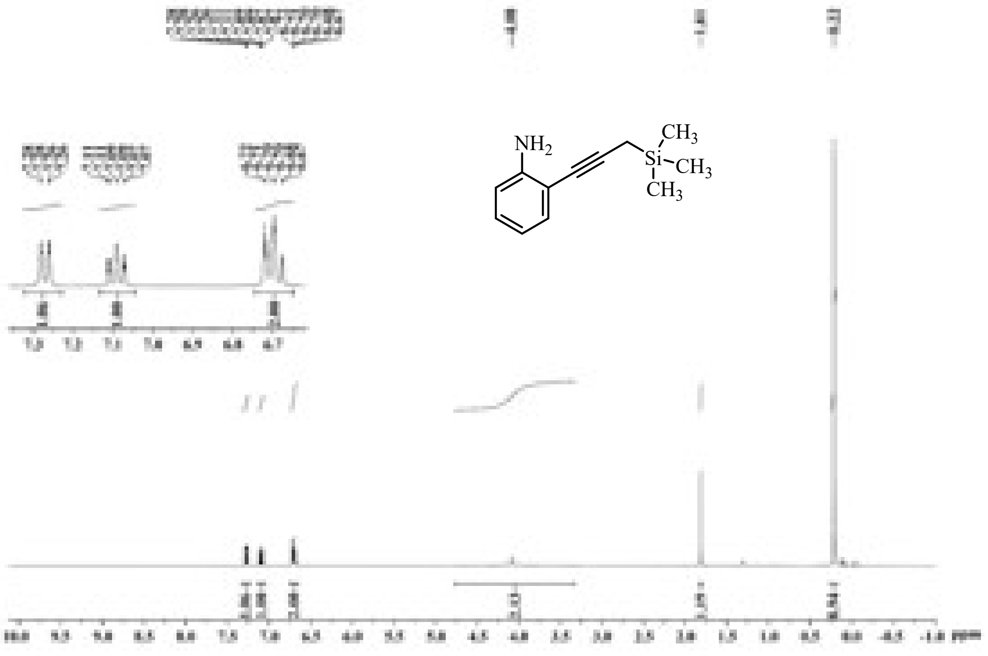

Figure S12. ¹H NMR spectrum of 3b

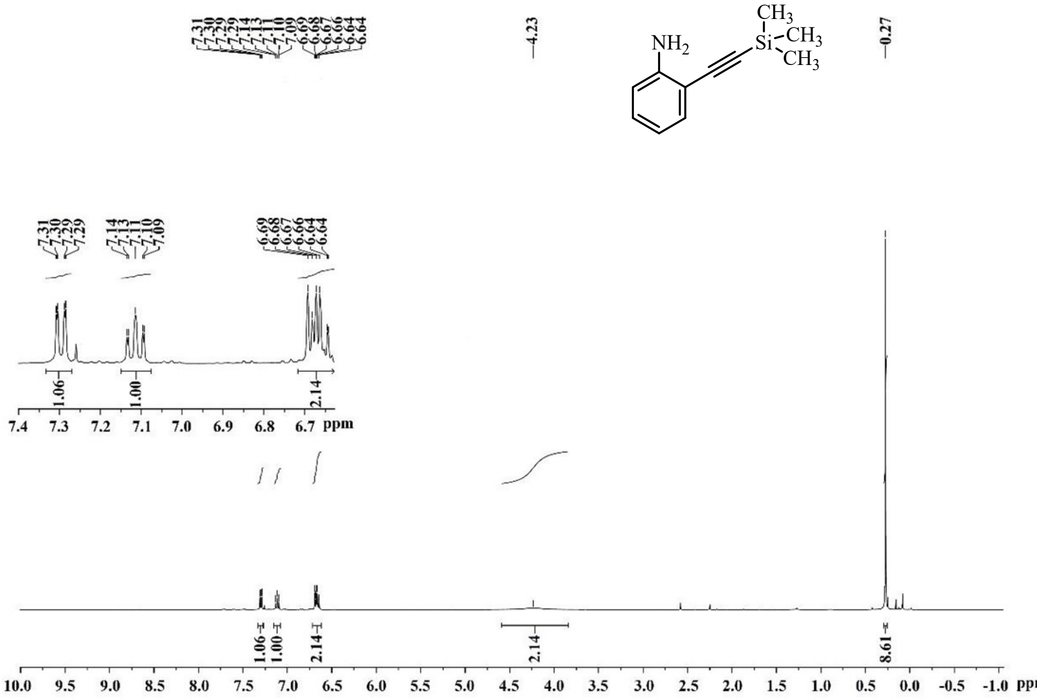

Figure S13. ^{1}H NMR spectrum of 3c

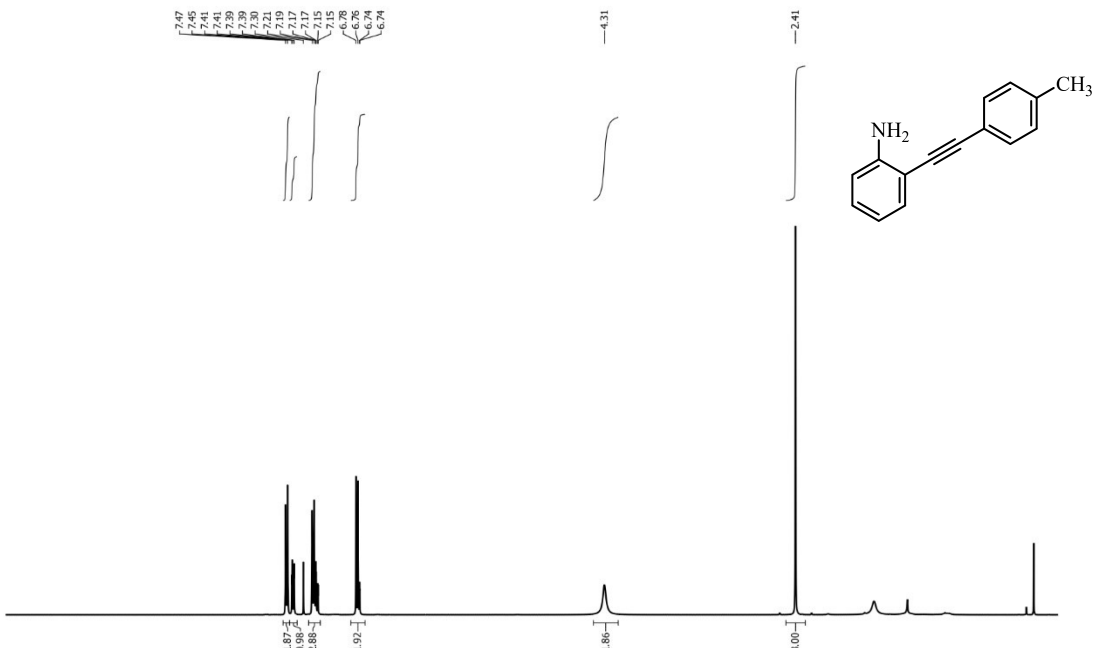

Figure S14. ^{1}H NMR spectrum of 3d

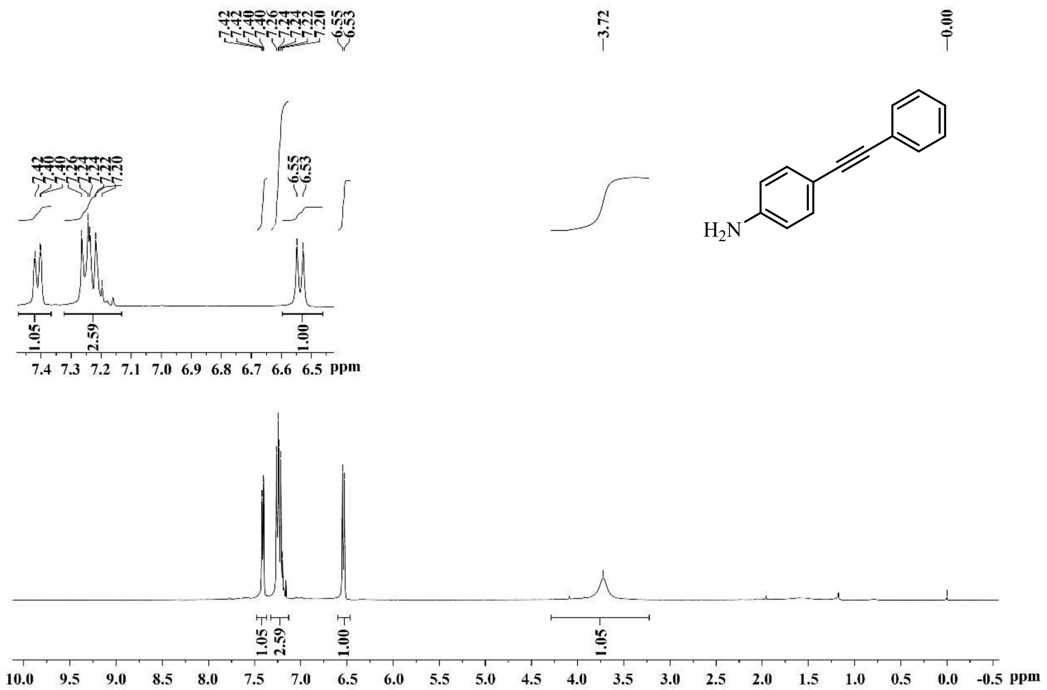

Figure S15. ^{1}H NMR spectrum of 3e

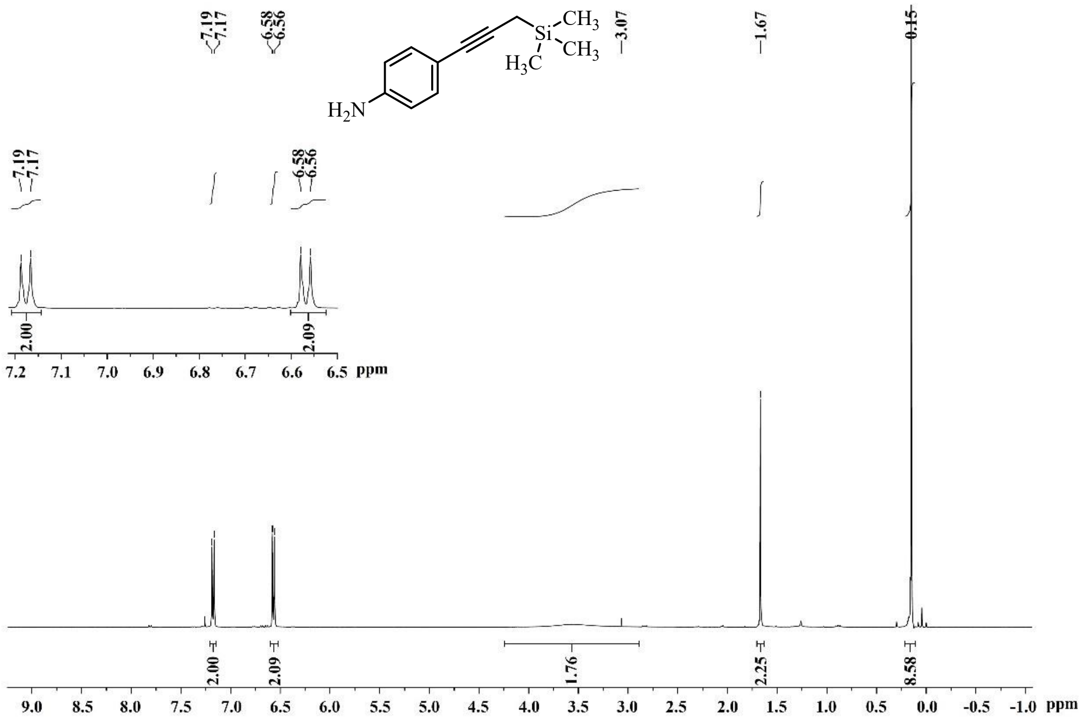

Figure S16. ^{1}H NMR spectrum of 3f

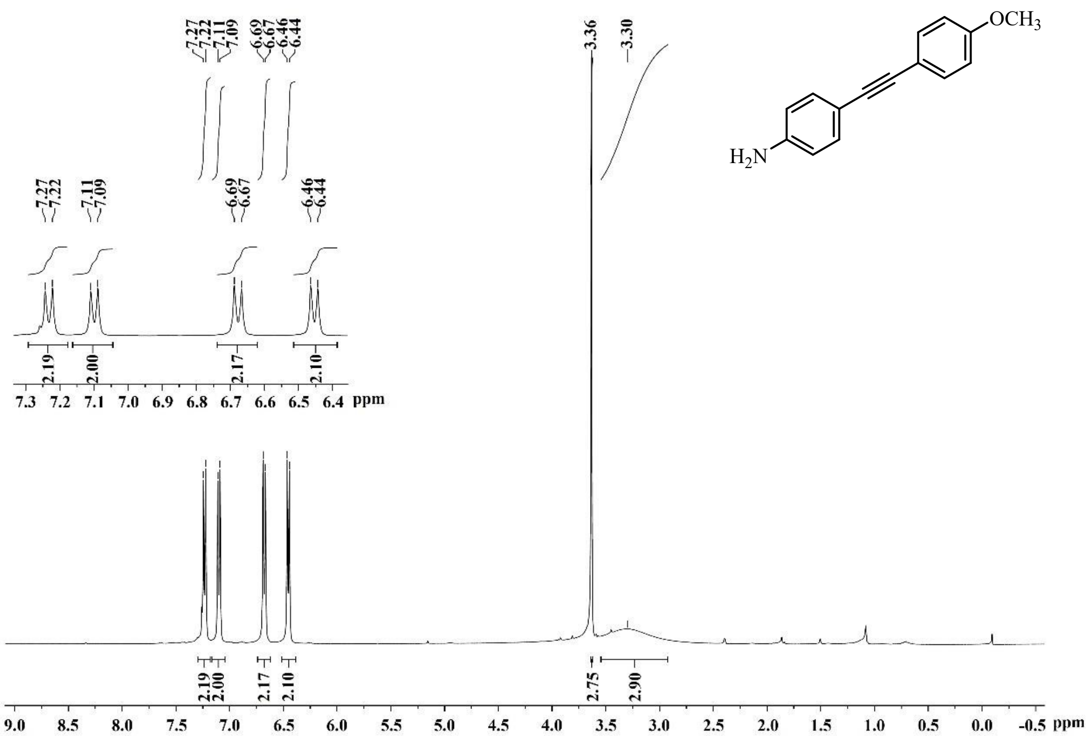

Figure S17. ^{1}H NMR spectrum of 3g

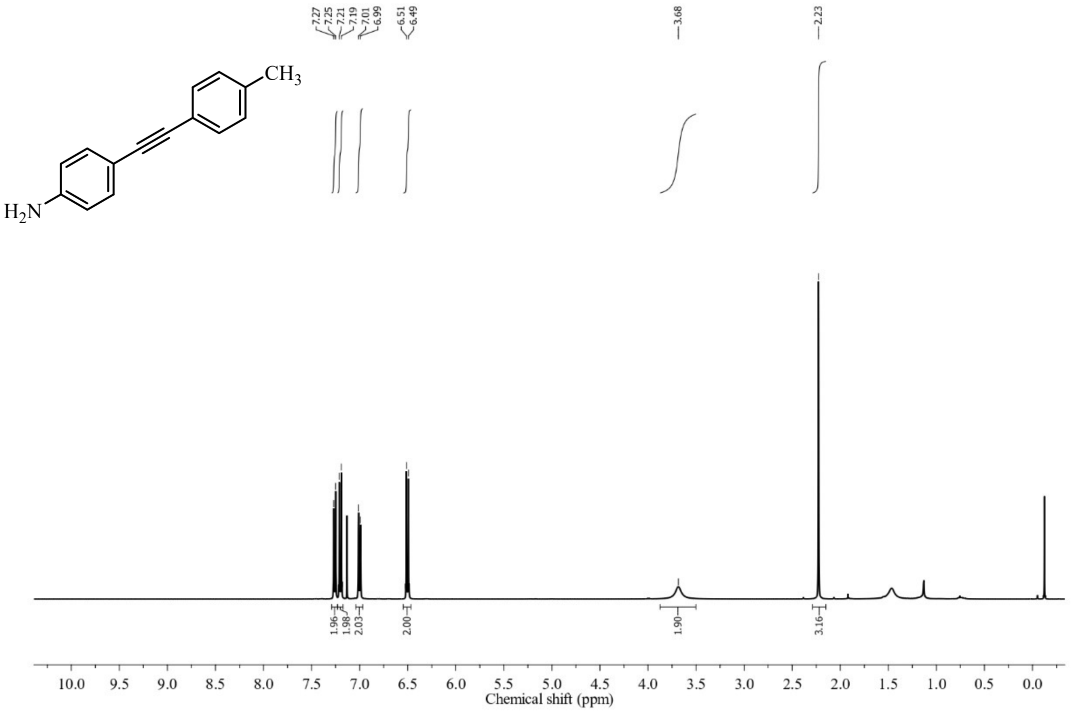

Figure S18. ^{1}H NMR spectrum of 3h

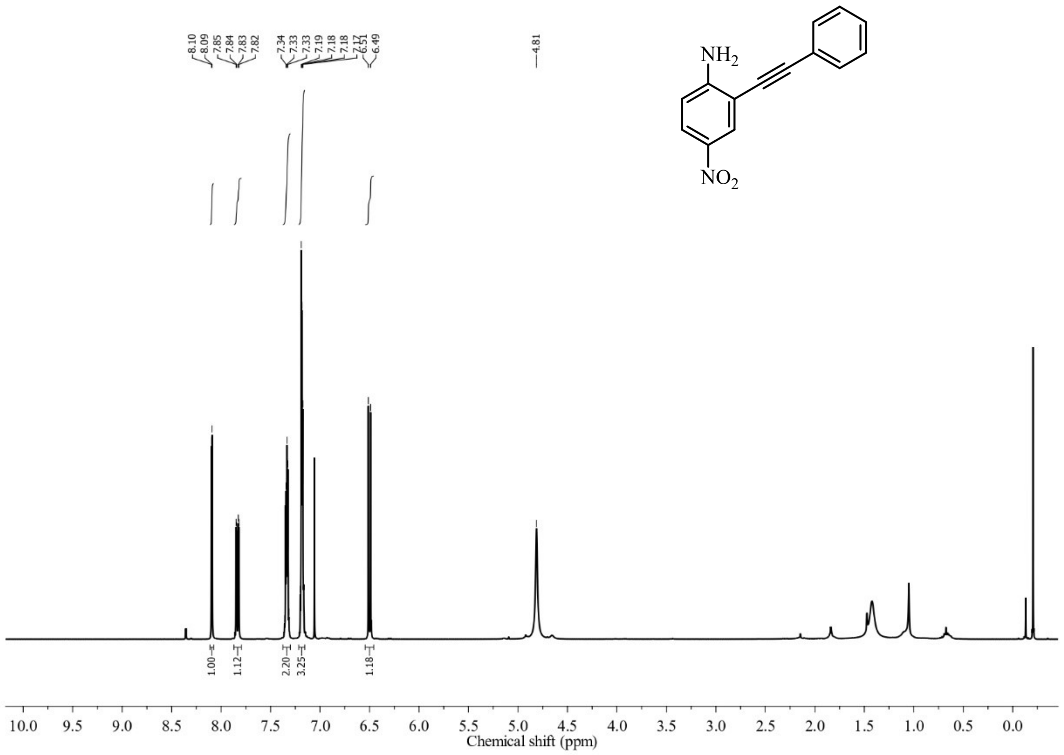

Figure S19. ^{1}H NMR spectrum of 3i

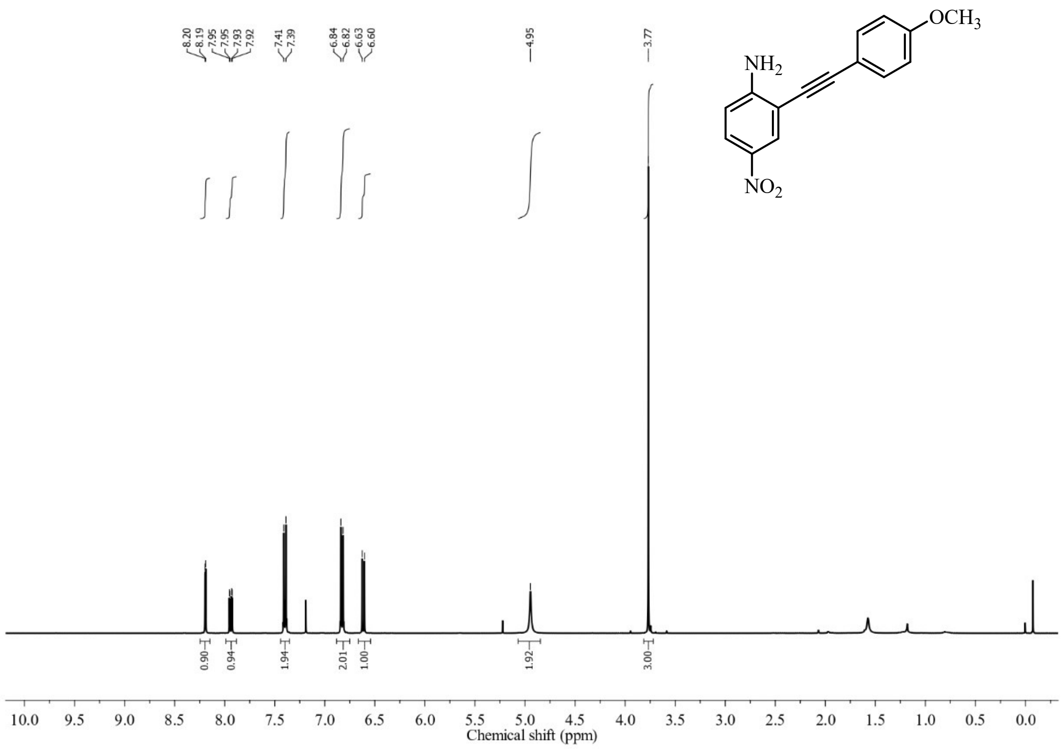

Figure S20. ^{1}H NMR spectrum of 3j

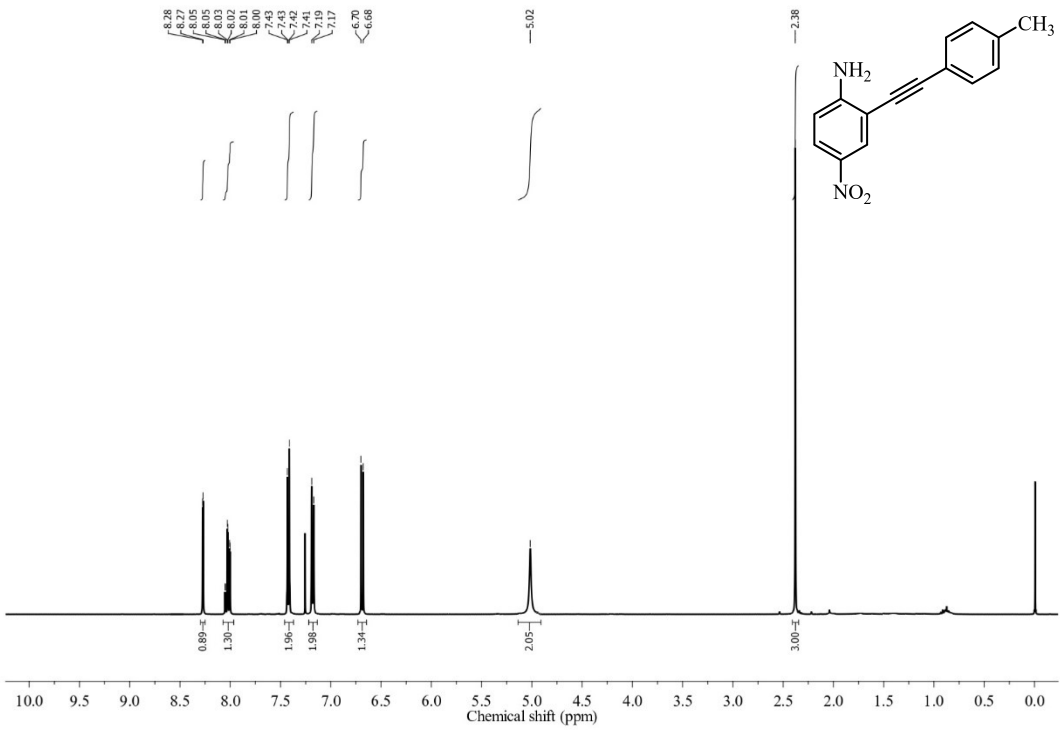

Figure S21. ^{1}H NMR spectrum of 3k

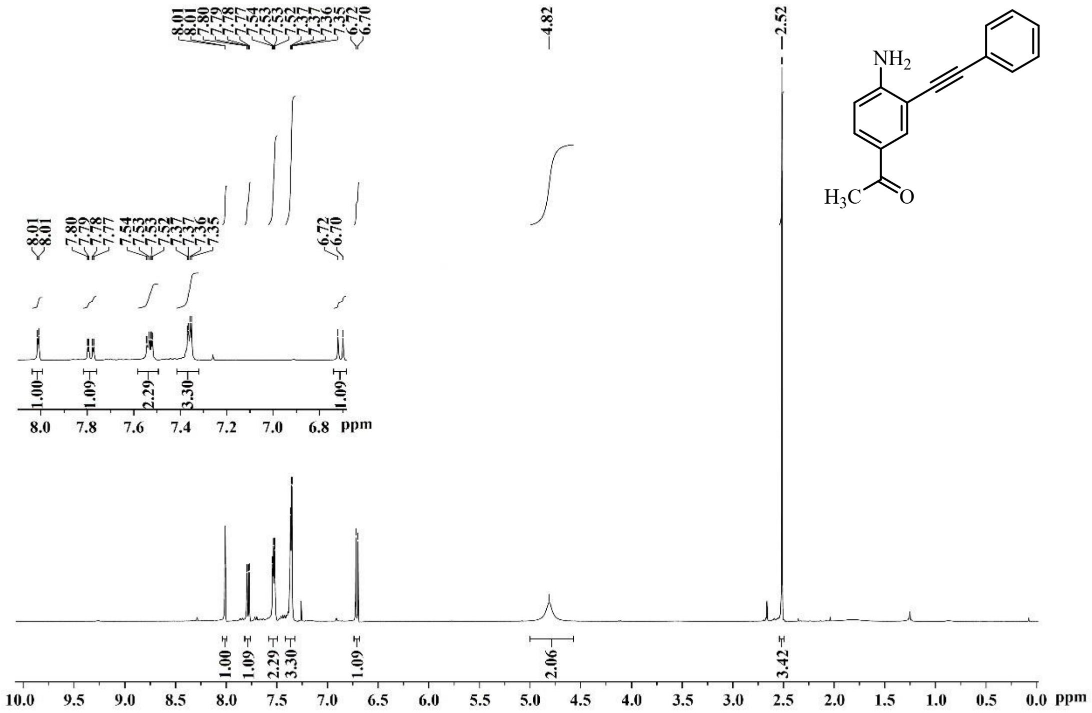

Figure S22. ^{1}H NMR spectrum of 3l

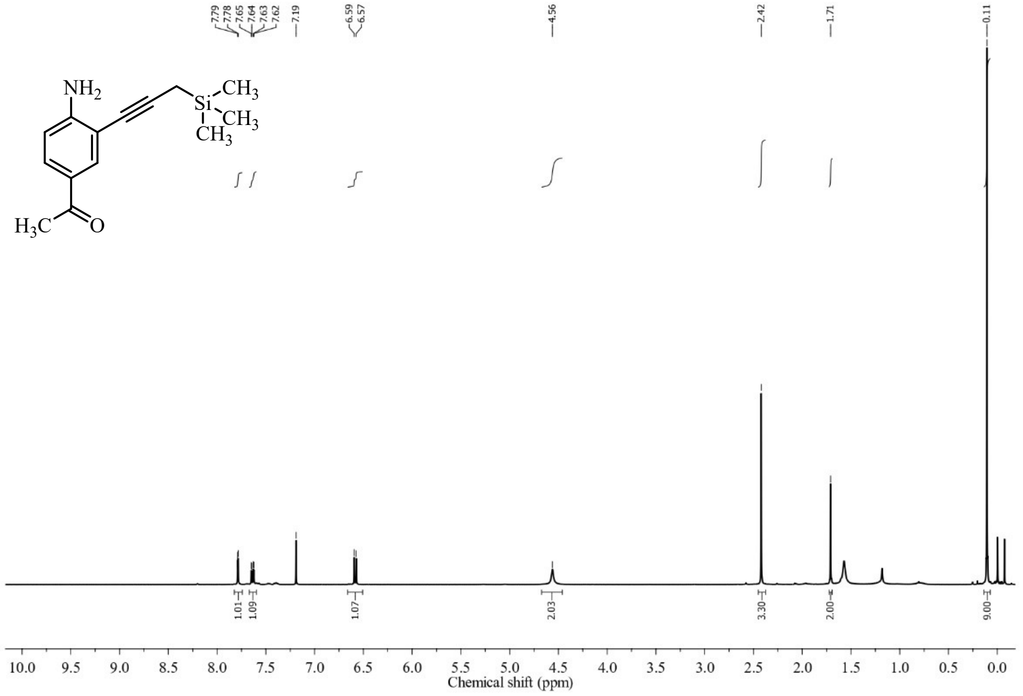

Figure S23. ^{1}H NMR spectrum of 3m

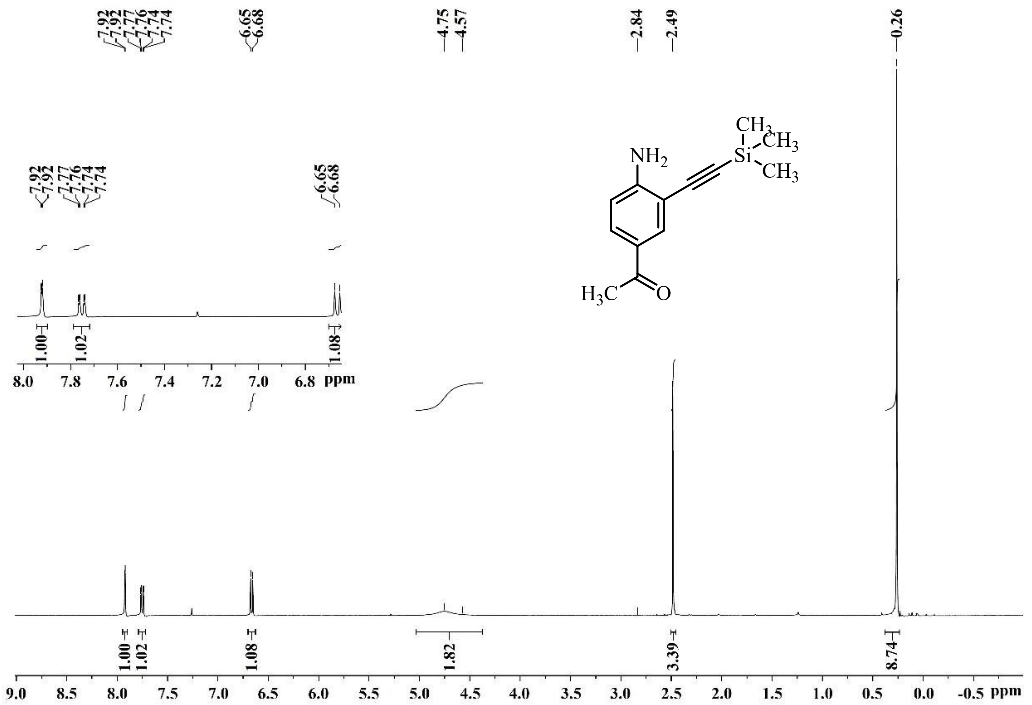

Figure S24. ^{1}H NMR spectrum of 3n

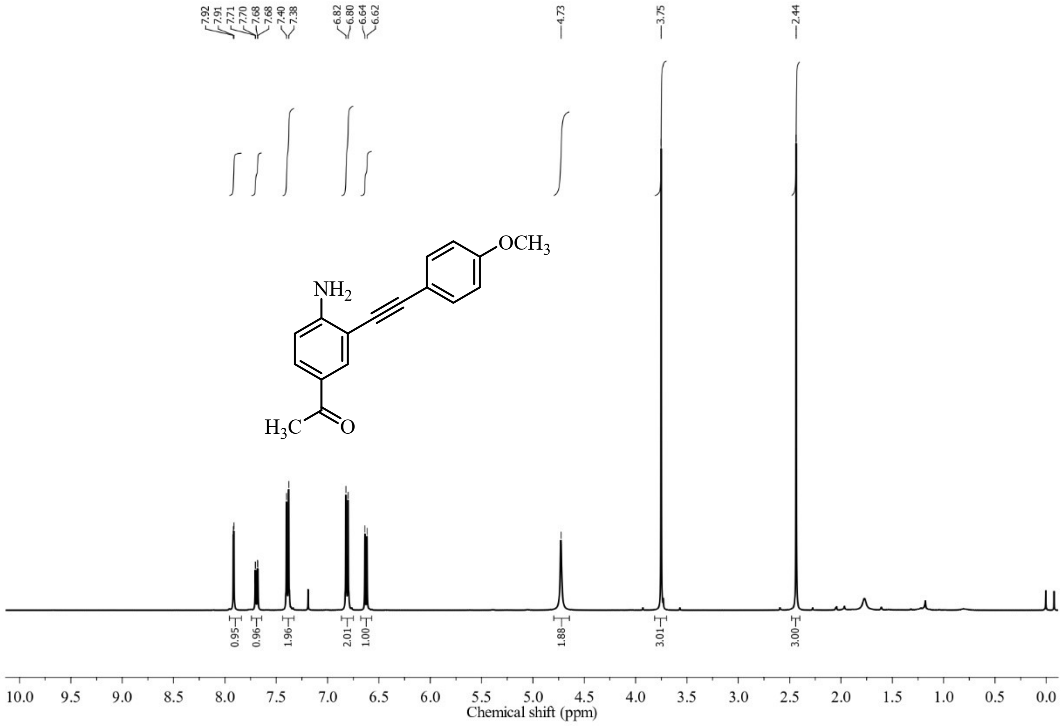

Figure S25. ^{1}H NMR spectrum of 3o

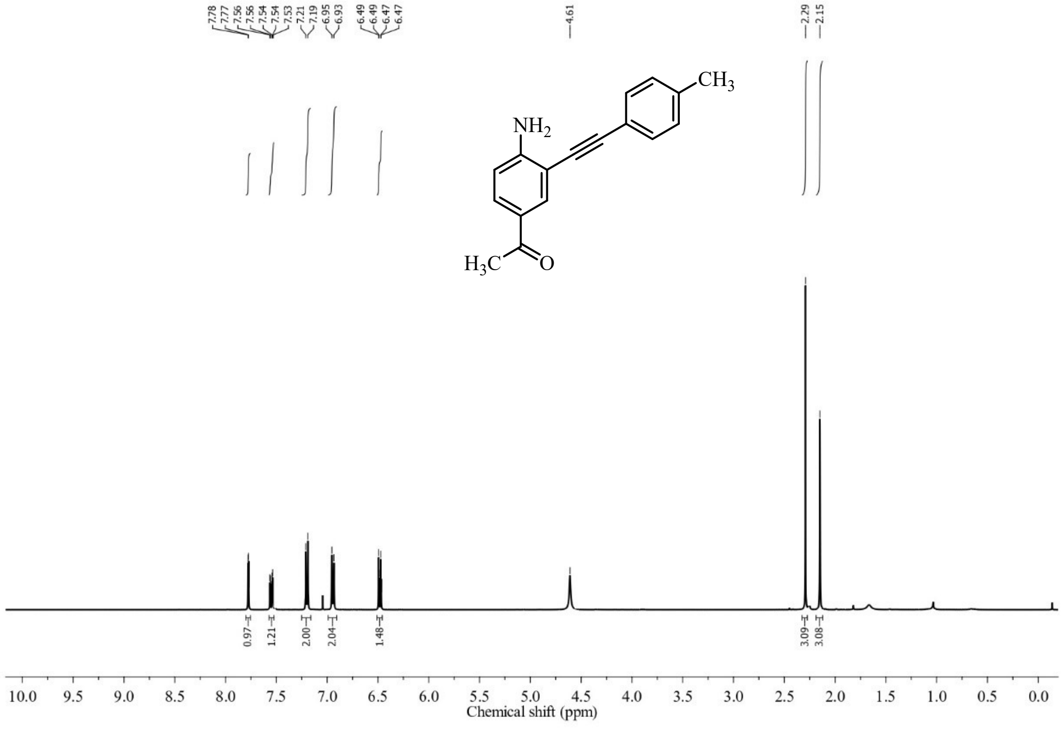

Figure S26. ^{1}H NMR spectrum of 3p

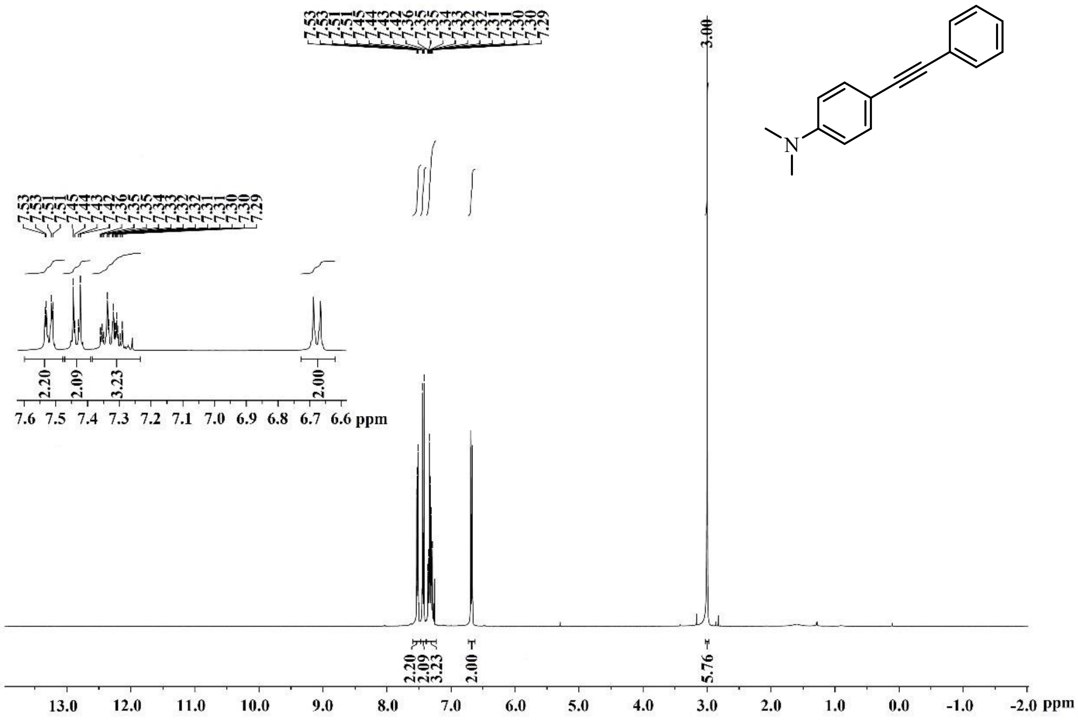

Figure S27. ^{1}H NMR spectrum of 3q

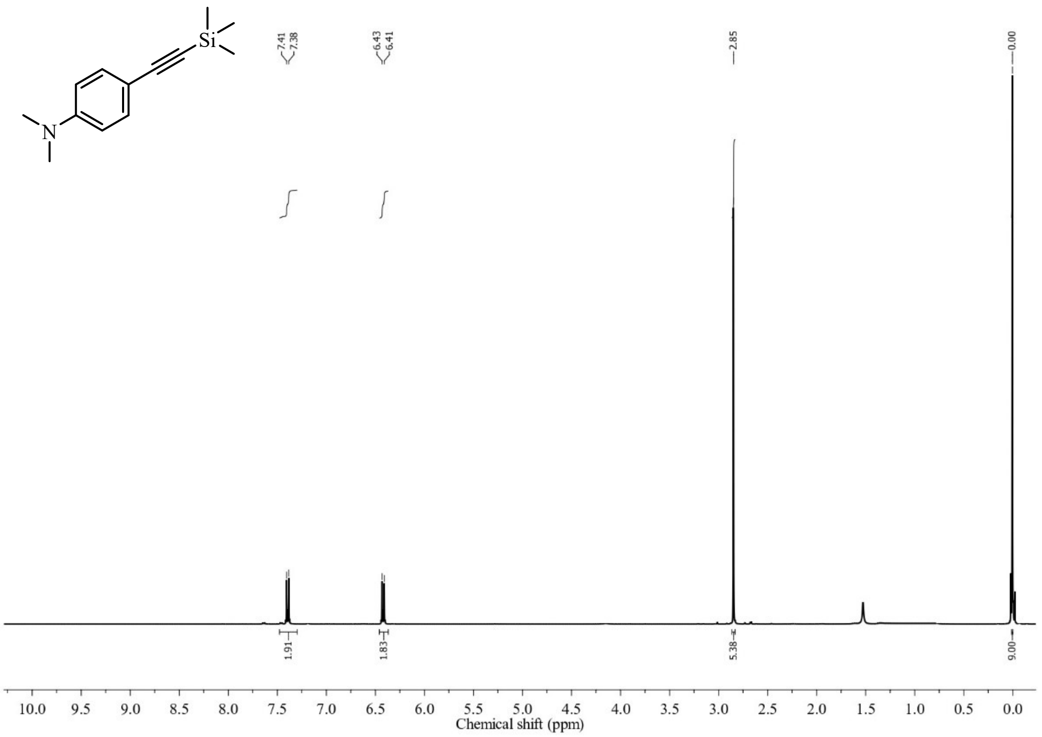

Figure S28. ^{1}H NMR spectrum of 3r

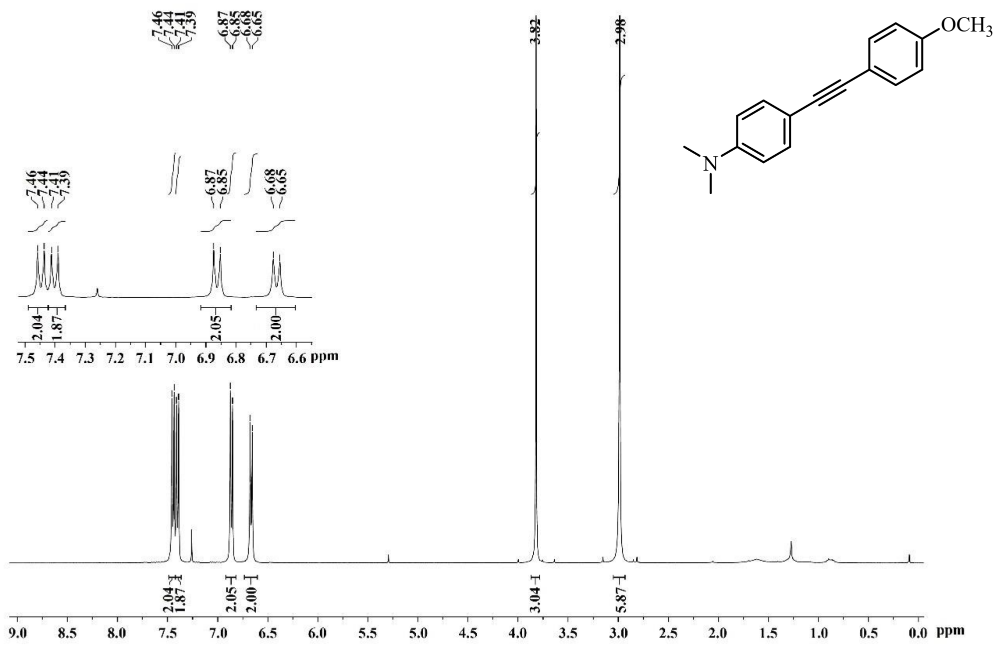

Figure S29. ^{1}H NMR spectrum of 3s

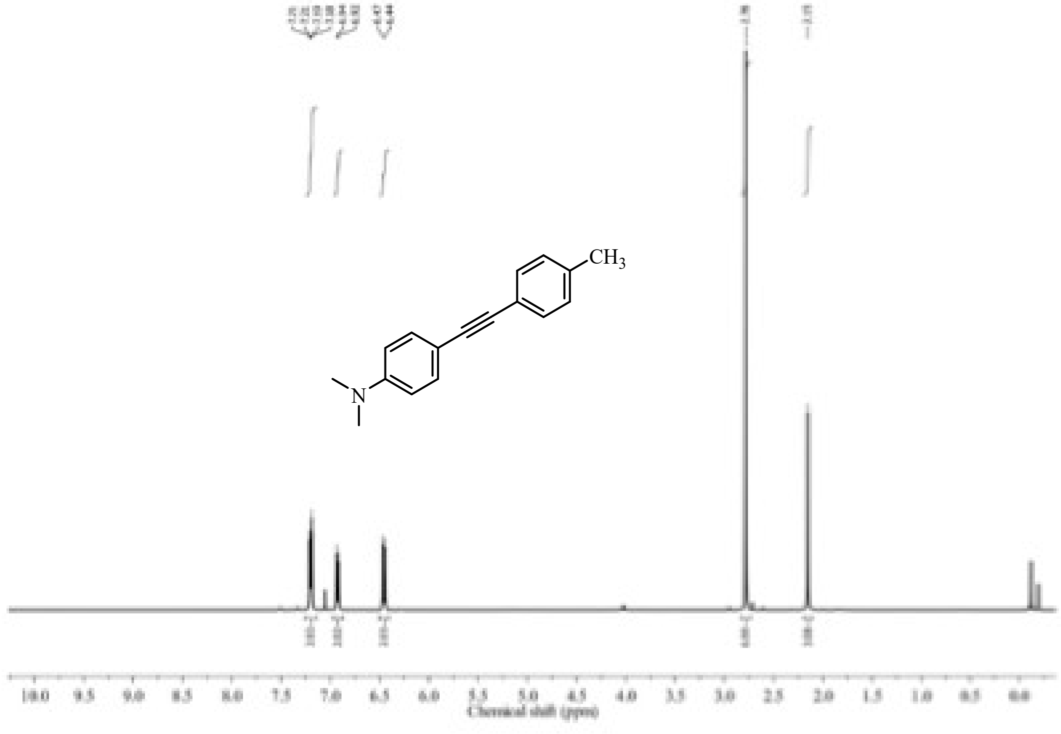

Figure S30. ^{1}H NMR spectrum of 3t

S8. ^{13}C NMR Spectra of Products

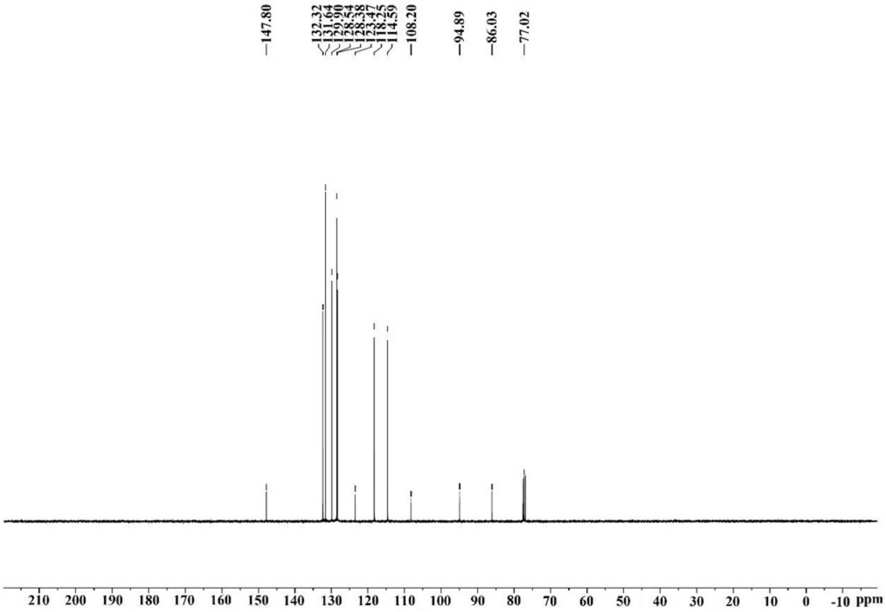

Figure S31. ^{13}C NMR spectrum of 3a

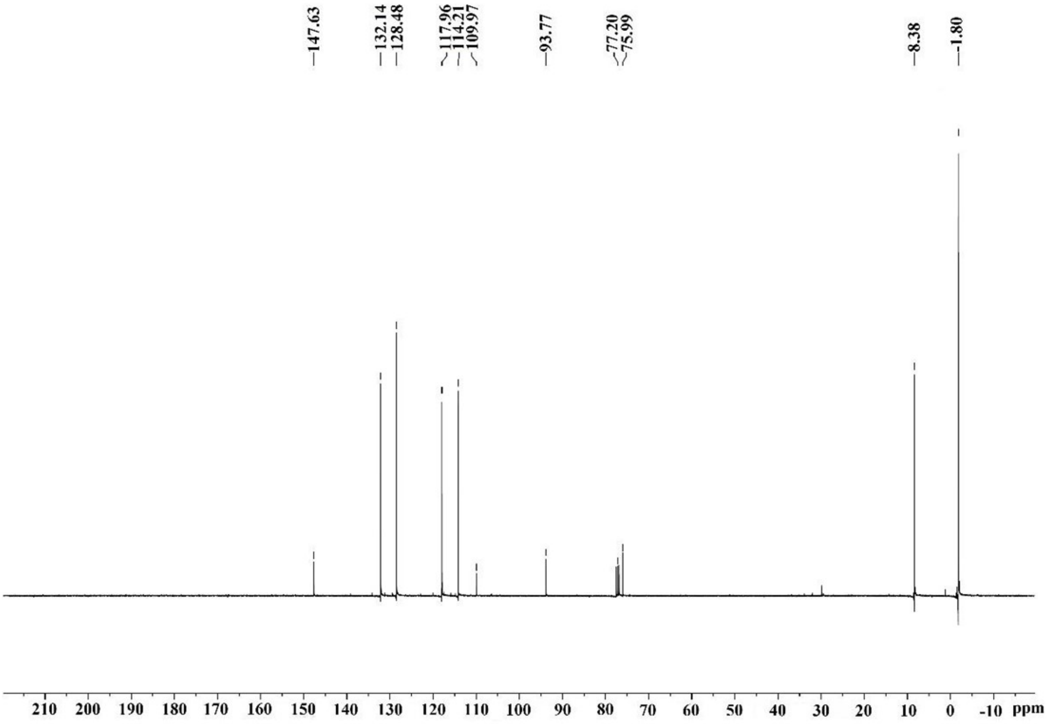

Figure S32. ^{13}C NMR spectrum of 3b

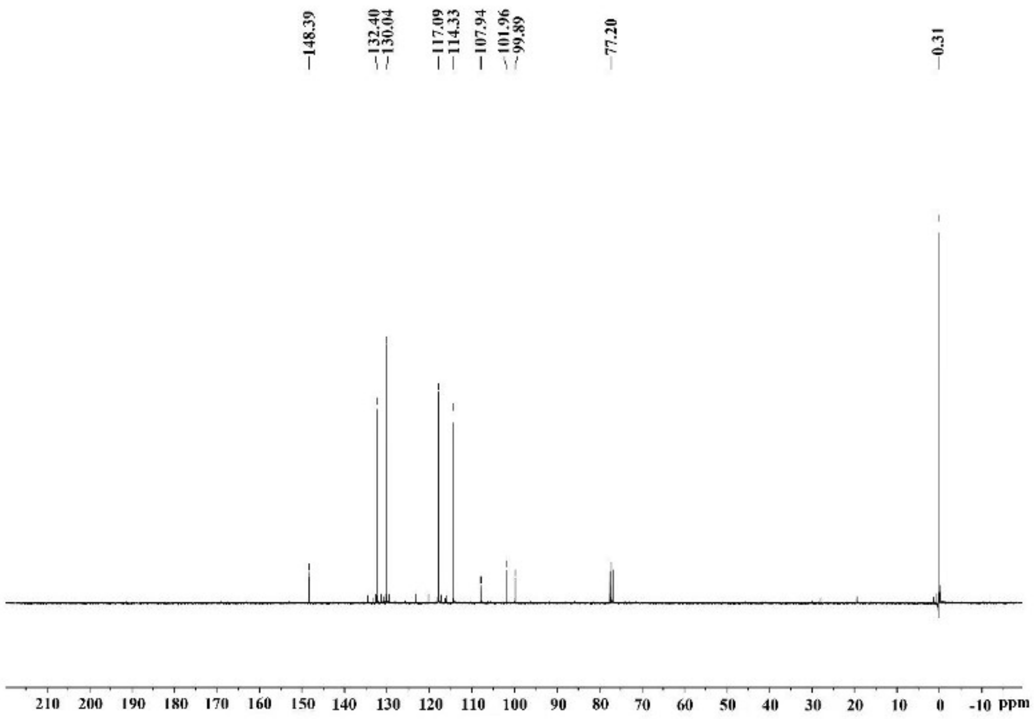

Figure S33. ^{13}C NMR spectrum of 3c

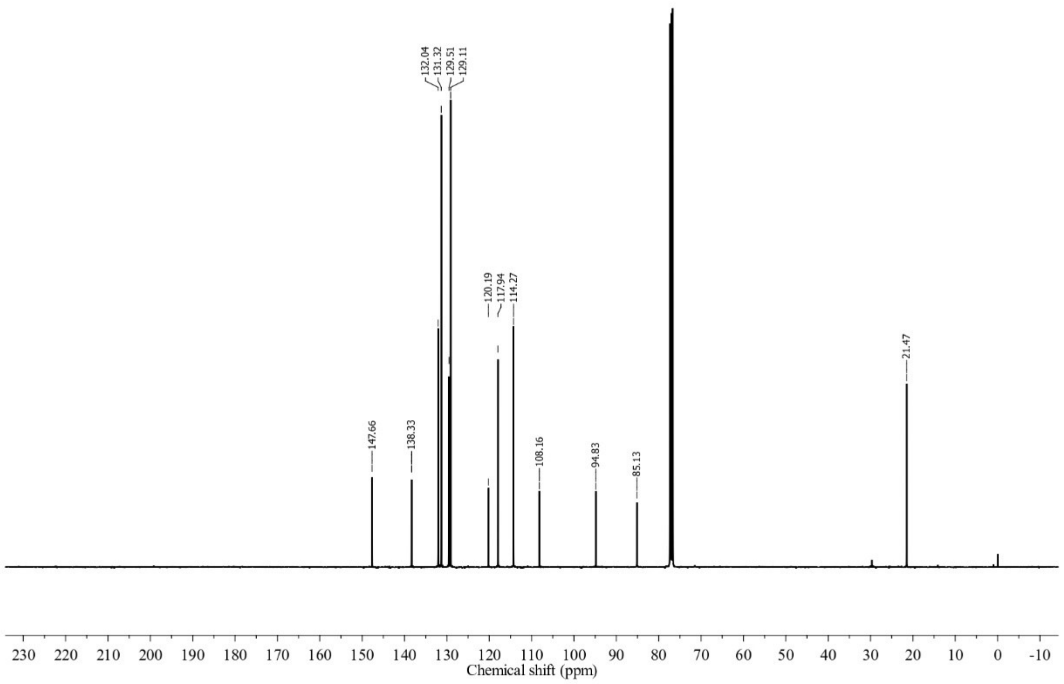

Figure S34. ^{13}C NMR spectrum of 3d

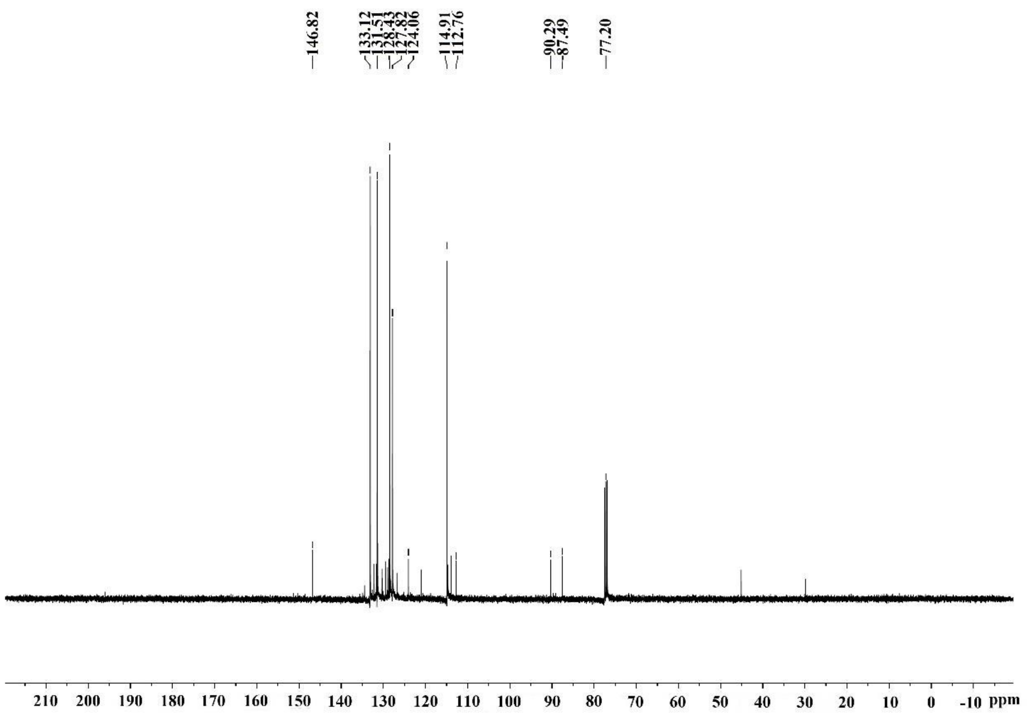

Figure S35. ^{13}C NMR spectrum of 3e

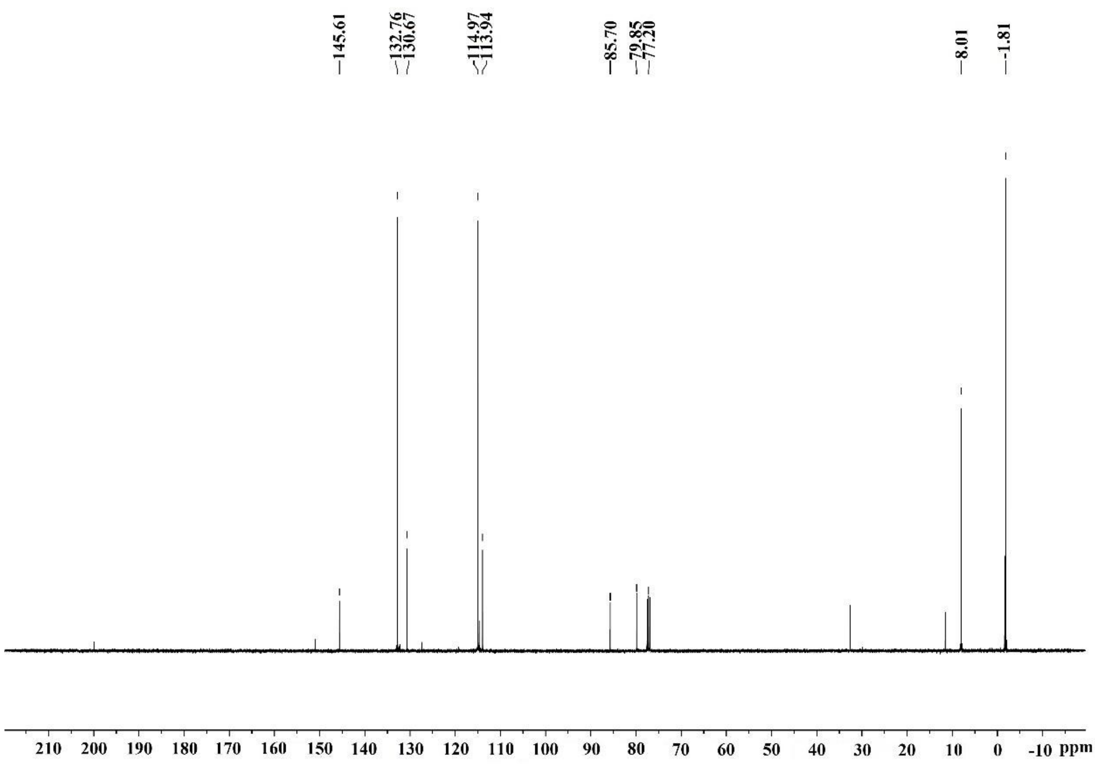

Figure S36. ^{13}C NMR spectrum of 3f

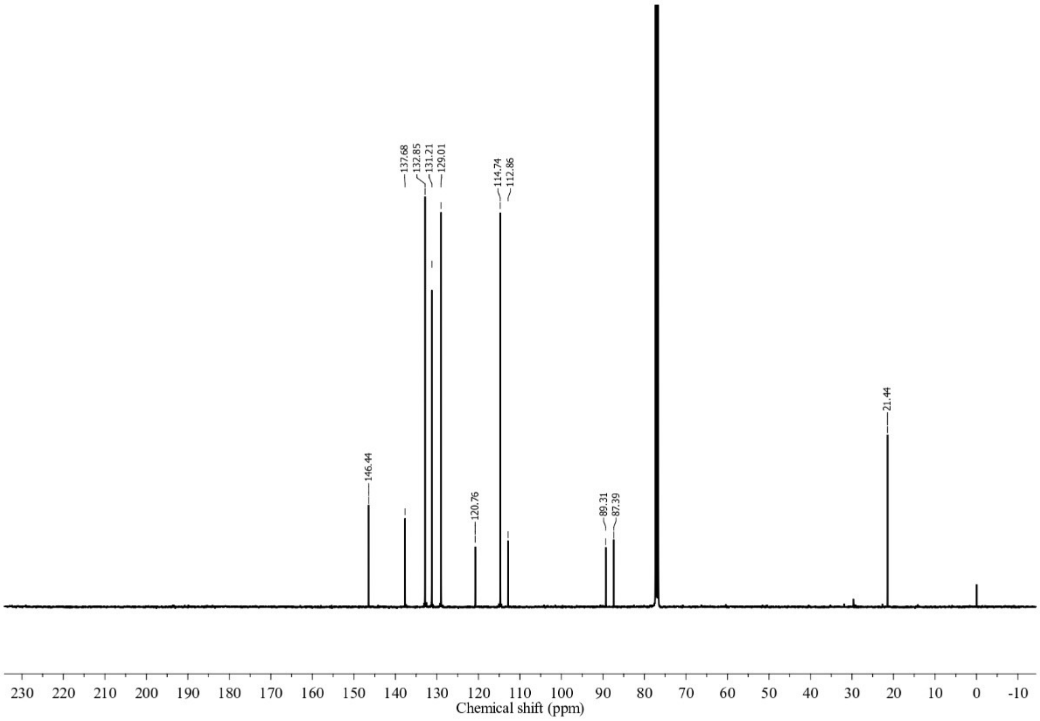

Figure S37. ^{13}C NMR spectrum of 3h

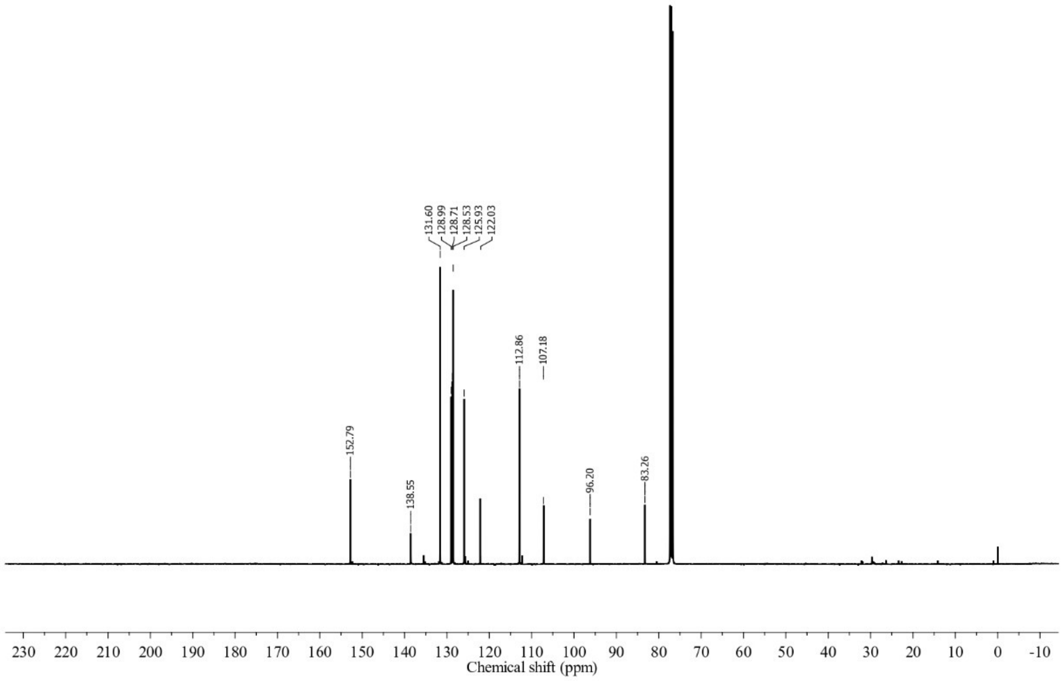

Figure S38. ^{13}C NMR spectrum of 3i

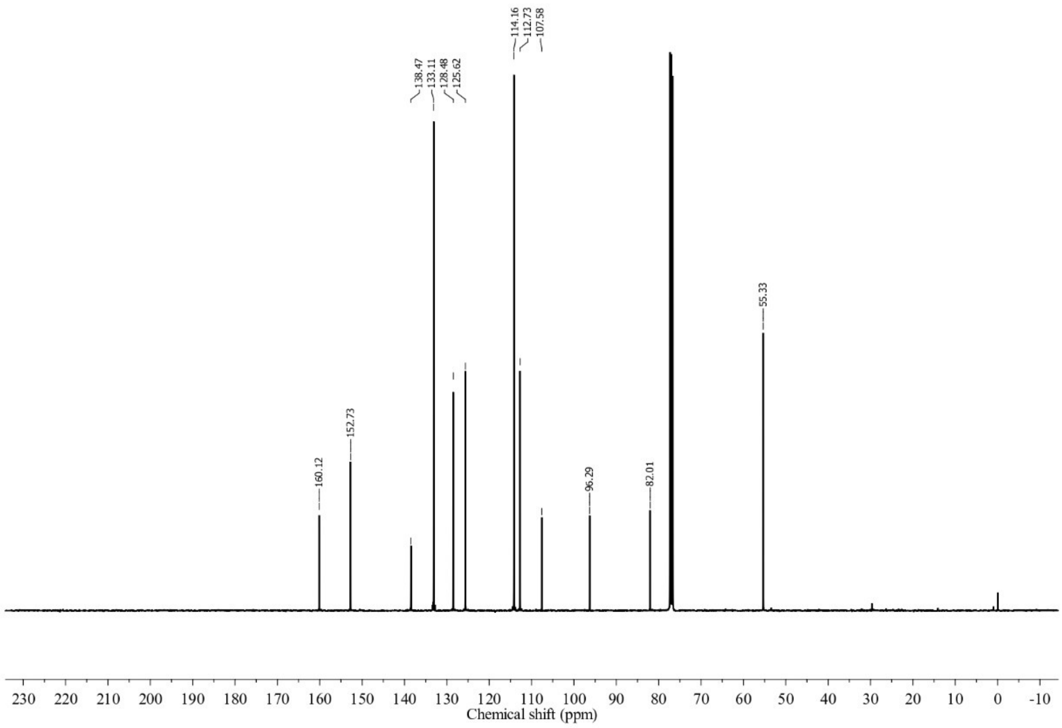

Figure S39. ^{13}C NMR spectrum of 3j

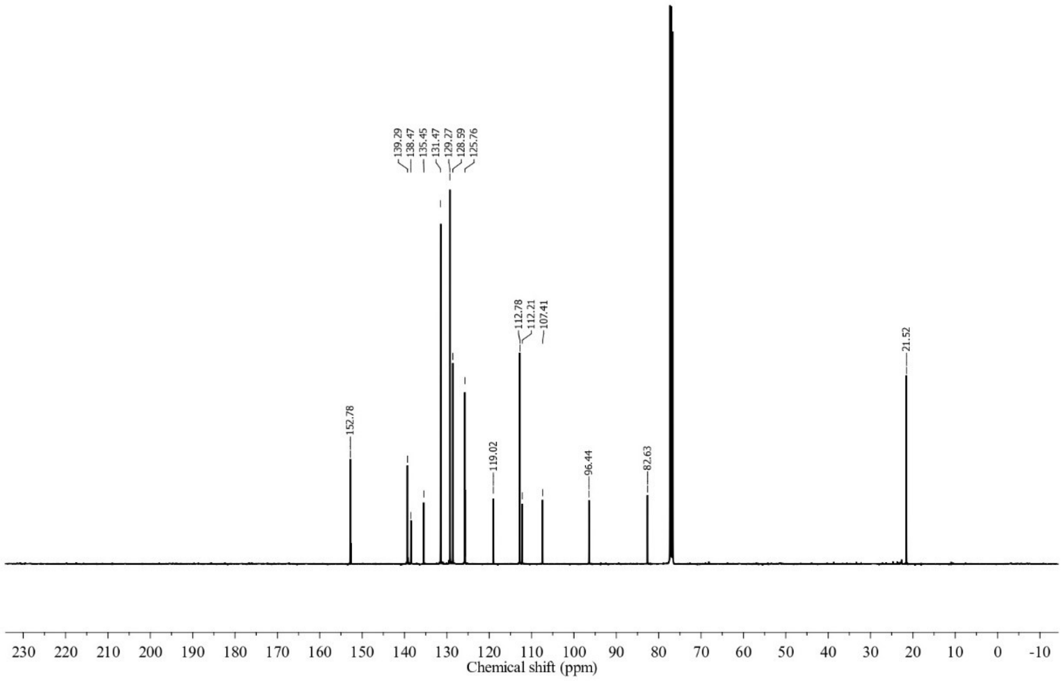

Figure S40. ^{13}C NMR spectrum of 3k

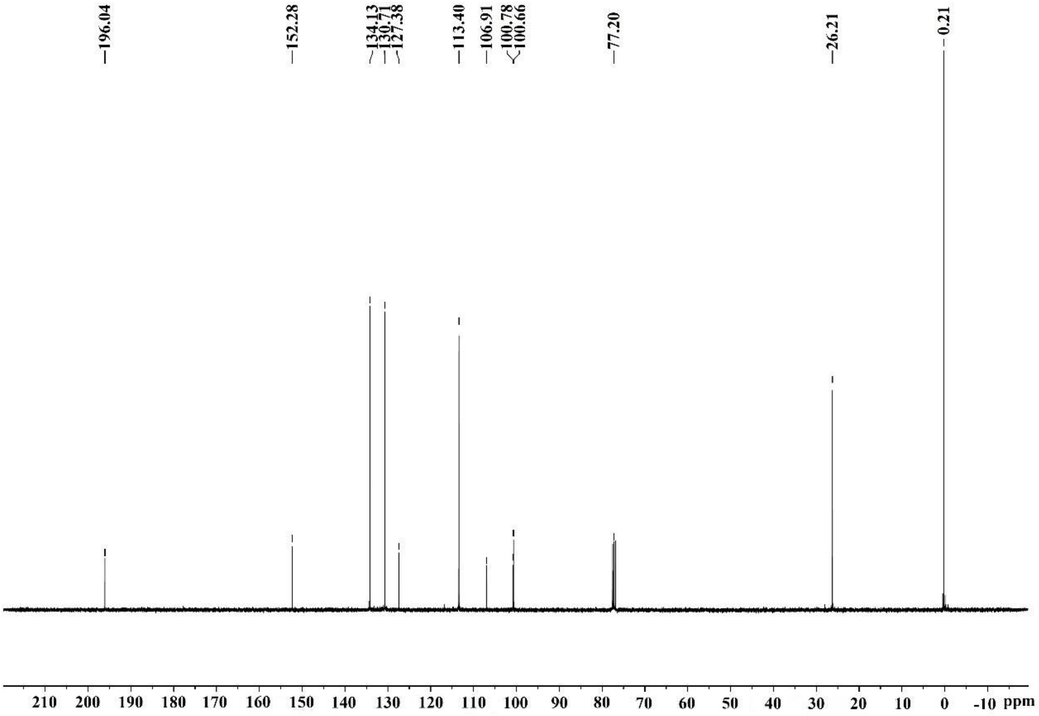

Figure S41. ^{13}C NMR spectrum of 3n

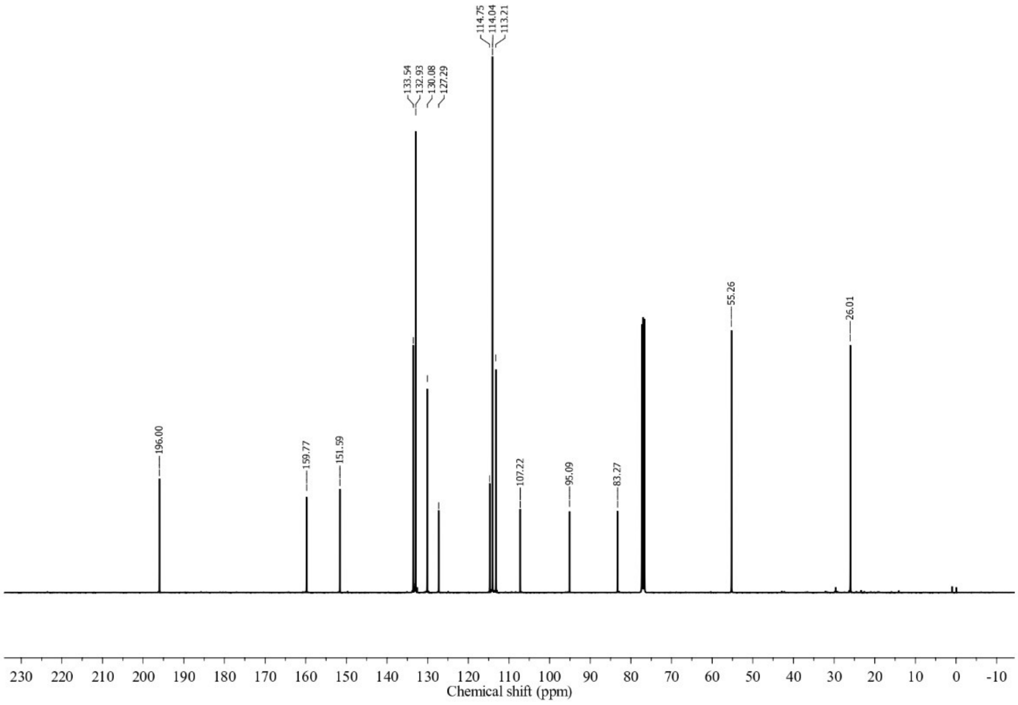

Figure S42. ^{13}C NMR spectrum of 3o

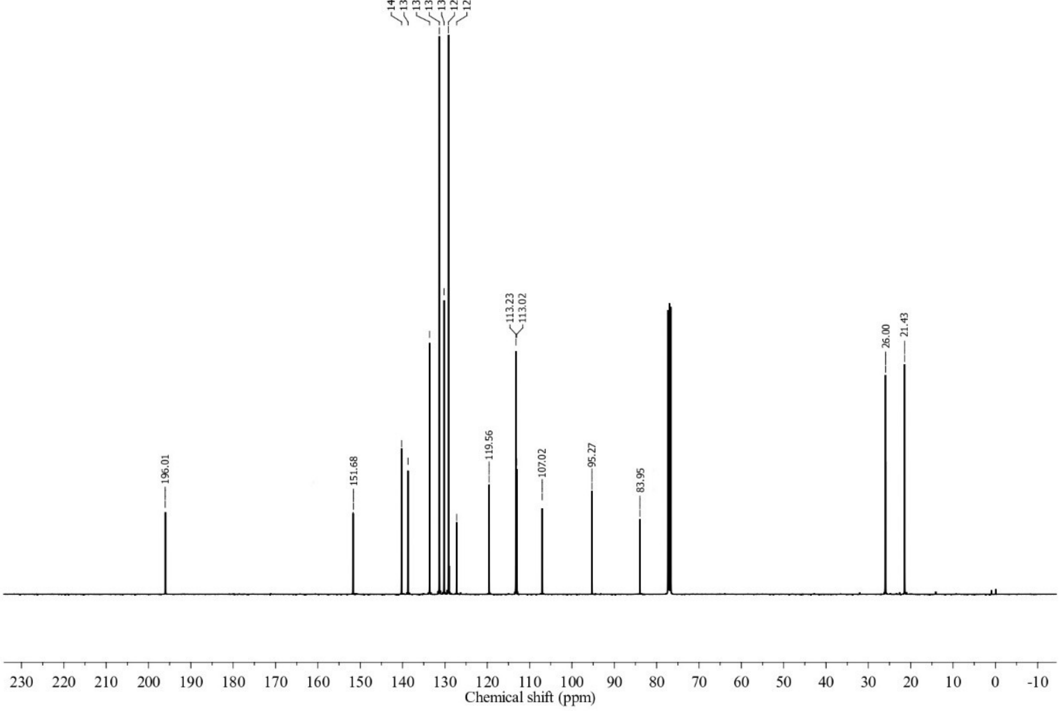

Figure S43. ^{13}C NMR spectrum of 3p

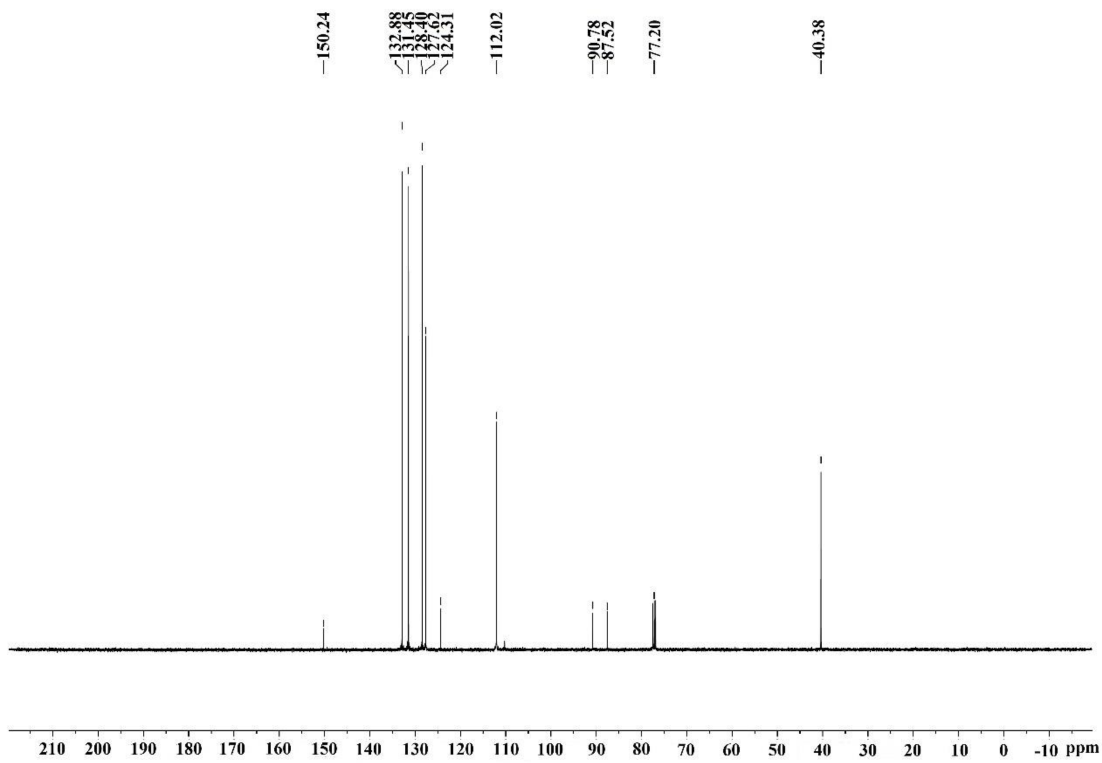

Figure S44. ^{13}C NMR spectrum of 3q

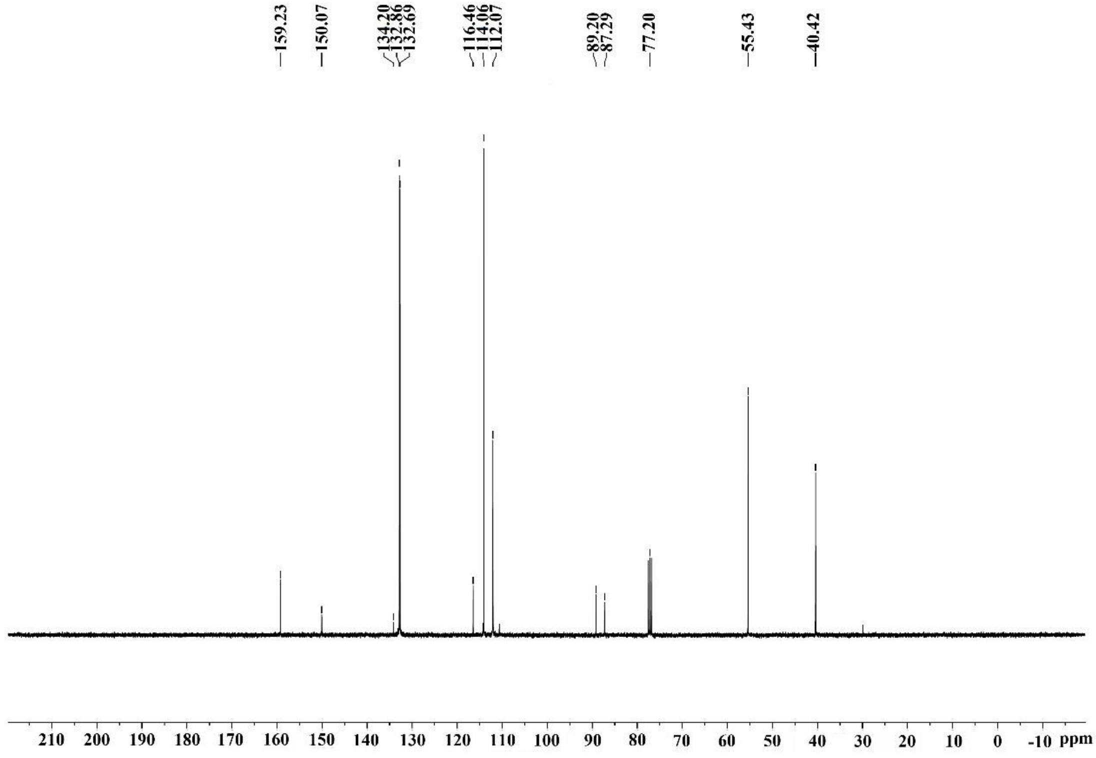

Figure S45. ^{13}C NMR spectrum of 3s

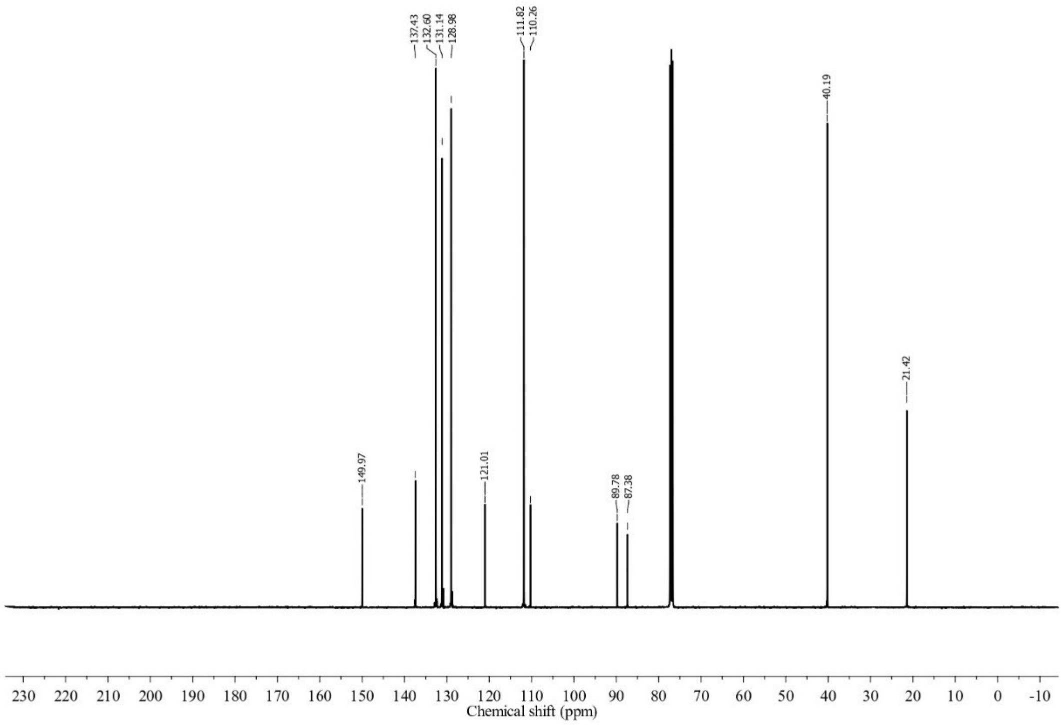

Figure S46. ^{13}C NMR spectrum of 3t

S9. HRMS spectra of selected products.

Mass Spectrum SmartFormula Report

Analysis Info

Analysis Name	D:\Data\Job\ESI\Job-HRMS 494 (pos).d	Acquisition Date	3/9/2026 12:39:26 PM
Method	tune_low_ pos.m	Operator	RU
Sample Name	3b	Instrument	micrOTOF 8213750.10411
Comment			

Acquisition Parameter

Source Type	ESI	Ion Polarity	Positive	Set Nebulizer	2.0 Bar
Focus	Not active			Set Dry Heater	200 °C
Scan Begin	50 m/z	Set Capillary	4500 V	Set Dry Gas	8.0 l/min
Scan End	2000 m/z	Set End Plate Offset	-500 V	Set Divert Valve	Waste

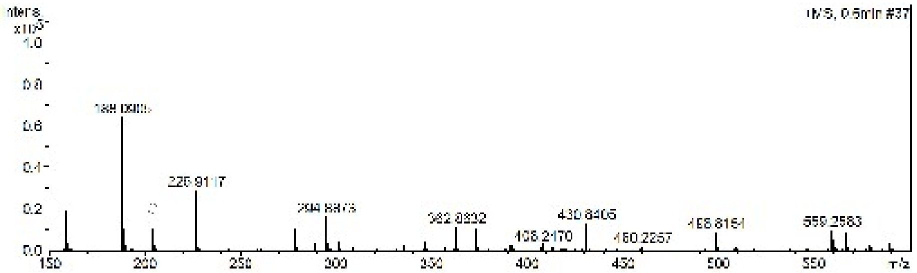

Meas. m/z	#	Ion Formula	m/z	|err| [mDa]	err [ppm]	Mean err [ppm]	rdb	N-Rule	e¯ Conf	mSigma
204.122201	1	C12H18NSi	204.120303	1.9	9.3	-9.5	5.5	ok	even	24.7

Figure S47. HRMS spectrum of 3b.

Mass Spectrum SmartFormula Report

Analysis Info

Analysis Name	D:\Data\Job\ESI\Job-HRMS 495 (pos).d	Acquisition Date	3/9/2026 12:43:35 PM
Method	tune_low_ pos.m	Operator	RU
Sample Name	3f	Instrument	micrOTOF 8213750.10411
Comment			

Acquisition Parameter

Source Type	ESI	Ion Polarity	Positive	Set Nebulizer	2.0 Bar
Focus	Not active			Set Dry Heater	200 °C
Scan Begin	50 m/z	Set Capillary	4500 V	Set Dry Gas	8.0 l/min
Scan End	2000 m/z	Set End Plate Offset	-500 V	Set Divert Valve	Waste

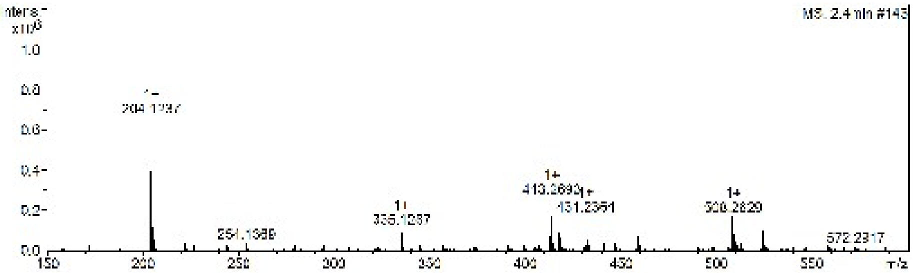

Meas. m/z	#	Ion Formula	m/z	|err| [mDa]	err [ppm]	Mean err [ppm]	rdb	N-Rule	e⁻ Conf	mSigma
204.123656	1	C12H18NSi	204.120303	3.4	16.4	-10.8	5.5	ok	even	16.1

Bruker Compass DataAnalysis 4.1 printed: 3/9/2026 5:25:49 PM by: RU 1 of 1

Figure S48. HRMS spectrum of 3f.

Mass Spectrum SmartFormula Report

**Analysis Info**		Acquisition Date	3/9/2026 5:10:48 PM
Analysis Name	D:\Data\Job\ESI\Job-HRMS 497 (pos).d		
Method	tune_low_ pos.m	Operator	RU
Sample Name	3m	Instrument	micrOTOF 8213750.10411
Comment			

Acquisition Parameter

Source Type	ESI	Ion Polarity	Positive	Set Nebulizer	2.0 Bar
Focus	Not active			Set Dry Heater	200 °C
Scan Begin	50 m/z	Set Capillary	4500 V	Set Dry Gas	8.0 l/min
Scan End	2000 m/z	Set End Plate Offset	-500 V	Set Divert Valve	Waste

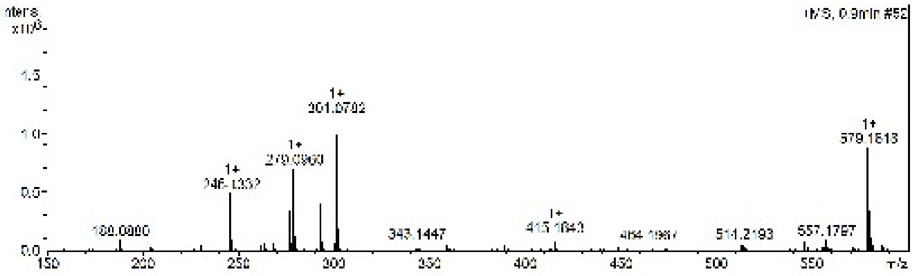

Meas. m/z	#	Ion Formula	m/z	|err| [mDa]	err [ppm]	Mean err [ppm]	rdb	N-Rule	e⁻ Conf	mSigma
246.133168	1	C14H20NOSi	246.130867	2.3	9.3	-8.7	6.5	ok	even	6.3

Figure S49. HRMS spectrum of 3m.

Mass Spectrum SmartFormula Report

Analysis Info

Analysis Name	D:\Data\Job\ESI\Job-HRMS 498 (pos).d	Acquisition Date	3/9/2026 12:50:06 PM
Method	tune_low_ pos.m	Operator	RU
Sample Name	3o	Instrument	micrOTOF 8213750.10411
Comment			

Acquisition Parameter

Source Type	ESI	Ion Polarity	Positive	Set Nebulizer	2.0 Bar
Focus	Not active			Set Dry Heater	200 °C
Scan Begin	50 m/z	Set Capillary	4500 V	Set Dry Gas	8.0 l/min
Scan End	2000 m/z	Set End Plate Offset	-500 V	Set Divert Valve	Waste

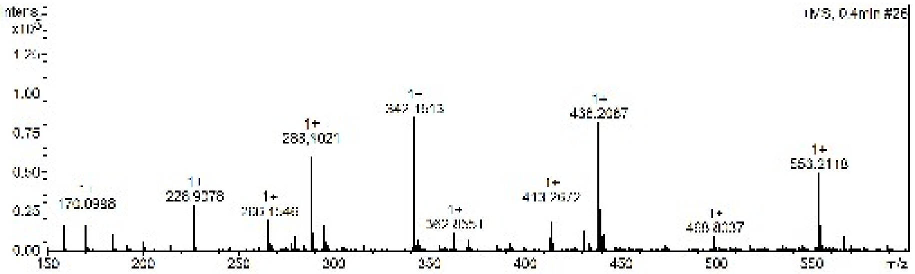

Meas. m/z	#	Ion Formula	m/z	|err| [mDa]	err [ppm]	Mean err [ppm]	rdb	N-Rule	e⁻ Conf	mSigma
288.102130	1	C17H15NNaO2	288.099499	2.6	9.1	-10.2	10.5	ok	even	3.0

Figure S50. HRMS spectrum of 3o.

Mass Spectrum SmartFormula Report

Analysis Info

Analysis Name	D:\Data\Job\ESI\Job-HRMS 499 (pos).d	Acquisition Date	3/9/2026 12:51:50 PM
Method	tune_low_ pos.m	Operator	RU
Sample Name	3p	Instrument	micrOTOF 8213750.10411
Comment			

Acquisition Parameter

Source Type	ESI	Ion Polarity	Positive	Set Nebulizer	2.0 Bar
Focus	Not active			Set Dry Heater	200 °C
Scan Begin	50 m/z	Set Capillary	4500 V	Set Dry Gas	8.0 l/min
Scan End	2000 m/z	Set End Plate Offset	-500 V	Set Divert Valve	Waste

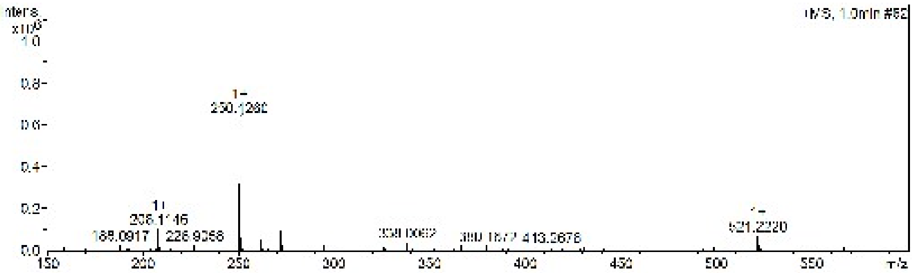

Meas. m/z	#	Ion Formula	m/z	|err| [mDa]	err [ppm]	Mean err [ppm]	rdb	N-Rule	e⁻ Conf	mSigma
250.125993	1	C17H16NO	250.122641	3.4	13.4	-12.9	10.5	ok	even	3.3

Bruker Compass DataAnalysis 4.1 printed: 3/9/2026 5:38:07 PM by: RU 1 of 1

Figure S51. HRMS spectrum of 3p.

S10. Literature References for Known Compounds

The spectral data of compounds 3a, 3c, 3d, 3e, 3g–3l, 3n, and 3q–3t were consistent with those reported in the literature. Literature references of known aryl-alkynyl aniline derivatives are summarised in Table S3.

Table S3. Literature references for previously reported compounds.

Entry	Compound	Literature reference
1	2-(Phenylethynyl)aniline (3a)	Priyadarshini, S.; Amal Joseph, P. J.; Srinivas, P.; Maheswaran, H.; Lakshmi Kantam, M.; Bhargava, S. *Tetrahedron Lett.*, 2011, **52**, 1615–1618. https://doi.org/10.1016/j.tetlet.2011.01.119
2	2-((Trimethylsilyl)ethynyl)aniline (3c)	Govindan, K.; Pasupuleti, R.; Ramalingam, A.; Chen, N.-Q.; Lin, Y.-C.; Lin, W.-Y. *Chem. Asian J.*, 2026, **21**, e00961. https://doi.org/10.1002/asia.202500961
3	2-(4-Methylphenylethynyl)aniline (3d)	Kabalka, G. W.; Wang, L.; Pagni, R. M. *Tetrahedron*, 2001, **57**, 8017–8028. https://doi.org/10.1016/S0040-4020(01)00774-8.

Entry	Compound	Literature reference
4	4-(Phenylethynyl)aniline (3e)	Karami, K.; Dehghani Najvani, S.; Haghighat Naeini, N.; Hervés, P., Chinese J. Catal., 2015, 36, 1047–1053. https://doi.org/10.1016/S1872-2067(15)60837-3
5	4-((4-Methoxyphenyl)ethynyl)aniline (3g)	C. Rosseinsky, J. Majimel, E. Fouquet, C. Delbecq, M. Boujtita, C. Aymonier and H. T. Nguyen-DucLong, *Chem. Eur. J.*, 2013, **19**, 12582–12589. https://doi.org/10.1002/chem.201300347
6	4-(*p*-Tolylethynyl)aniline (3h)	S. N. Jadhav, A. S. Kumbhar, S. S. Mali, C. K. Hong and R. S. Salunkhe, *New J. Chem.*, 2015, **39**, 2477–2486. https://doi.org/10.1039/C4NJ02025A
7	4-Nitro-2-(phenylethynyl)aniline (3i)	A. R. Katritzky, Y. Zhang, J. K. Singh and S. K. Sharma, *J. Org. Chem.*, 2023, **88**, 6736–

Entry	Compound	Literature reference
		6749. https://doi.org/10.1021/acs.joc.3c00027
8	2-((4-Methoxyphenyl)ethynyl)-4-nitroaniline (3j)	A. R. Katritzky, Y. Zhang, J. K. Singh and S. K. Sharma, *J. Org. Chem.*, 2023, **88**, 6736–6749. https://doi.org/10.1021/acs.joc.3c00027
9	4-Nitro-2-(*p*-tolylethynyl)aniline (3k)	J. S. Ward, P. R. Raithby and A. S. K. Hashmi, *Org. Biomol. Chem.*, 2015, **13**, 346–356. https://doi.org/10.1039/C5OB00121A
10	1-(4-Amino-3-(phenylethynyl)phenyl)ethan-1-one (3l)	A. Carpita and A. Ribecai, *Tetrahedron Lett.*, 2009, **50**, 6877–6881. https://doi.org/10.1016/j.tetlet.2009.09.135
11	1-(4-Amino-3-((trimethylsilyl)ethynyl)phenyl)ethan-1-one (3n)	A. Ikeda, M. Omote, K. Kusumoto, M. Komori, A. Tarui, K. Sato and A. Ando, *Org. Biomol. Chem.*, 2016, **14**, 2127–2133. https://doi.org/10.1039/C5OB02558C

**Entry**	**Compound**	**Literature reference**
12	*N,N*-Dimethyl-4-(phenylethynyl)aniline (3q)	M. Schilz and H. Plenio, *J. Org. Chem*., 2012, **77,** 2798–2807. https://doi.org/10.1021/jo202644g
13	*N,N*-Dimethyl-4-((trimethylsilyl)ethynyl)aniline (3r)	A. Elangovan, Y.-H. Wang and T.-I. Ho, *Org. Lett*., 2003, **5**, 1841–1844. https://doi.org/10.1021/ol034320+
14	4-((4-Methoxyphenyl)ethynyl)-*N,N*-dimethylaniline (3s)	Z. Jin, Q. Li, M. Zhu, Y. Zhang, X. Yan and X. Zhou, *RSC Adv*., 2025, 15, 7826–7831. https://doi.org/10.1039/D5RA00357A
15	*N,N*-Dimethyl-4-(*p*-tolylethynyl)aniline (3t)	B. Liang, M. Dai, J. Chen and Z. Yang, *J. Org. Chem*., 2005, **70**, 391–393, https://doi.org/10.1021/jo048599z
